# Functional plasticity of *Capsicum annuum* var. *glabriusculum* through multiple traits

**DOI:** 10.1093/aobpla/plac017

**Published:** 2022-05-05

**Authors:** Alberto Jiménez-Leyva, Jesús Orozco-Avitia, Aldo Gutiérrez, Georgina Vargas, Esteban Sánchez, Ezequiel Muñoz, Martín Esqueda

**Affiliations:** Centro de Investigación en Alimentación y Desarrollo, Carretera Gustavo Enrique Astiazarán Rosas No. 46, Col. La Victoria, Hermosillo, Sonora C.P. 83304, México; Centro de Investigación en Alimentación y Desarrollo, Carretera Gustavo Enrique Astiazarán Rosas No. 46, Col. La Victoria, Hermosillo, Sonora C.P. 83304, México; Centro de Investigación en Alimentación y Desarrollo, Carretera Gustavo Enrique Astiazarán Rosas No. 46, Col. La Victoria, Hermosillo, Sonora C.P. 83304, México; Centro de Investigación en Alimentación y Desarrollo, Carretera Gustavo Enrique Astiazarán Rosas No. 46, Col. La Victoria, Hermosillo, Sonora C.P. 83304, México; Centro de Investigación en Alimentación y Desarrollo, Av. 4ta Sur 3820, Fracc. Vencedores del Desierto, Delicias, Chihuahua C.P. 33089, México; Centro de Investigación en Alimentación y Desarrollo, Av. 4ta Sur 3820, Fracc. Vencedores del Desierto, Delicias, Chihuahua C.P. 33089, México; Centro de Investigación en Alimentación y Desarrollo, Carretera Gustavo Enrique Astiazarán Rosas No. 46, Col. La Victoria, Hermosillo, Sonora C.P. 83304, México

**Keywords:** C_3_ plants, ecophysiology, phenology, photosynthesis, Sonoran Desert

## Abstract

The diversity of functional traits still has not been studied enough in model plant species, even less so in little-known species. This experiment was carried out under the extreme heat of Sonoran Desert, using shading nets and under conditions where the availability of water and nutrients was not a stress factor. We evaluated how the low, intermediate and high sunlight regimes impact survival and promote multiple alterations on phenological and ecophysiological response of cultivated *Capsicum annuum* var. *glabriusculum* plants. Extremely warm temperatures promoted a high heat sum in degrees days throughout plants development. Most plants grown in high sunlight regimes did not survive; under intermediate sunlight regimes survival was high and plants developed vegetative and reproductively; but under low sunlight regimes plants survival was high; however, they developed just vegetatively. Photosynthetic response to light suggests that plants are physiologically acclimated to low and intermediate irradiance, whereas the CO_2_ assimilation curves suggest contrasting photosynthetic capacity traits. Under the intermediate sunlight regimes, plants strengthened their performance through multiple functional traits (e.g. CO_2_ and water diffusion traits, photosynthetic capacity, respiration, among others). Consequently, their biomass gain was faster and proportionally higher by 76 % with an investment of 14 % in fruits development. The principal components analysis extracted the main explanatory functional traits: photosynthetic nitrogen allocation, stomatal limitation, mesophyll conductance, Rubisco maximum carboxylation velocity, among others. In conclusion, phenological response and multiple functional traits determine plants acclimation to sunlight regimes and extremely warm temperatures in short term.

## Teaser

*Capsicum annuum*var. *glabriusculum*plants showed a contrasting phenological response and survival rate; under high sunlight regimes, there was no flower and fruit production in 5 % of surviving plants. In contrast, under intermediate sunlight regimes, plants showed vegetative and reproductive growth with an 86 % survival rate. In the low sunlight regimes, the plants developed vegetatively without flowers and fruits with a survival rate of 91 %. Plants also exhibited contrasting traits in the CO_2_and water diffusional efficiency, photosynthetic capacity and proportional limitation in stomata and mesophyll in response to sunlight regimes.

## Introduction

The variability of plants’ functional traits in response to environmental shifts is largely determined by their phenotypic and ecophysiological plasticity traits; therefore, in different environments and among plants species, the ability of genotypes to produce different phenotypes is the main strategy for their adaptation and evolution in the short and long term (Valladares *et al.* 2014). The sunlight regimes and air temperature thresholds (together with other complementary cues such as the vapour pressure deficit, rainfalls, CO_2_, soil biotic and abiotic traits) are the main drivers of plants phenological transitions and ecophysiological responses (Wadgymar *et al.* 2018; Fernández-Marín *et al.* 2020). For a robust analysis of phenological and ecophysiological traits either within individual species or among different species, microclimatic data sets are needed to provide realistic forecasts of plants’ fate under climate change pressures (Halbritter *et al.* 2020; Kattge *et al.* 2020). For such purposes, one of the main requirements is the tracking of environmental conditions, which implies recording mainly the sunlight regimes and air temperature thresholds, but also as many variables as possible during evaluations (e.g. CO_2_, vapour pressure deficit, rainfalls frequency/amount, irrigation/fertilization frequency and amount, and many others) (Poorter *et al.* 2012a, b, 2016).

Measurements and descriptions must accurately reflect multiple functional traits related to plants behaviour, e.g. phenological development, photosynthetic traits, mineral nutrition and a long list of parameters (Poorter *et al.* 2015, 2016, 2019). The availability of such data sets has broad implications not only for the prediction of productivity (either in individual, populations or communities) but also because is the basic requirement for comparison purposes through meta-analysis focused on phenological events, the carbon feedbacks and patterns of biomass allocation (Niinemets *et al.* 2015; Halbritter *et al.* 2020). Current meta-analysis about the functional equilibrium model shows that plants significantly change the allometric distribution of their biomass (fruits, leaves, stems, roots) in response to sunlight regimes and temperatures (Poorter *et al.* 2012c, 2015, 2019). However, still, there are multiple knowledge gaps behind such a model, since meta-analyses clearly show that functional plasticity behind plants phenological and ecophysiological responses still has not been studied enough in model species, even less so in little-known species (Flexas and Gago 2018; Niinemets 2018). Furthermore, the current global estimates of leaves traits in response to sunlight and temperatures are biased due to the wide plasticity existing within and between plant species (Keenan and Niinemets 2016; Niinemets 2018). Currently, there is scientific consensus that basic research about the impact of environmental conditions at a local level on plants’ phenological and ecophysiological performance will provide reference data sets aimed to reveal the different functional plasticity attributes (Poorter *et al.* 2019; Kattge *et al.* 2020).

This survey combines various research methods applied in C_3_ species (Martins *et al.* 2014; Poorter *et al.* 2016; Sun *et al.* 2014; Sharkey 2016). We focused on a woody, perennial and deciduous shrub: *Capsicum annuum* var. *glabriusculum*, a little-studied C_3_ species considered as the wild genetic ancestor of all varieties of domesticated and cultivated peppers worldwide (*C. annuum*), as well as a global priority species for *in situ* conservation programmes (Castañeda-Álvarez *et al.* 2016). Under the extreme heat of Sonoran Desert, we conducted an experiment where we used shading nets for plants cultivation under different sunlight, and in conditions where water and nutrient availability was not a stress factor. We addressed the following research question: Do the low, intermediate and high sunlight regimes drive a shift in survival rate and multiple alterations on the phenological and ecophysiological behaviour of cultivated plants? We hypothesized that under extremely warm temperatures, cultivated plants in low, intermediate and high sunlight regimes exhibit a positive survival rate, contrasting phenotypic attributes and multiple acclimation traits.

## Materials and Methods

### Experimental site and modification of sunlight regimes to differentially shade plants

This experiment was conducted under the extremely warm climate from Sonoran Desert (Hermosillo, Sonora, México; 29.128426 LN, −110.906437 LW). The historical trend of climatological conditions at the study site is described in **Supporting Information A**. For this experiment, we cultivated plants under the horticultural shading nets set to provide low, intermediate and high sunlight regimes (treatments). The daily photosynthetically active photons flux density (i.e. PPFD = sunlight regimes in mol m^−2^ day^−1^) was recorded throughout plants development by using a WatchDog™ ministation.

### Seeds germination, acclimation, transplanting, soil traits and growth conditions

This experiment lasted 157 days. Seeds were sowed in germination trays towards mid-spring. After 49 days plants were transferred to wet soil contained in big pots and they were distributed in the following sunlight regimes: low (*n* = 36), intermediate (*n* = 36) and high (*n* = 36). After transplantation, plants developed in the treatments throughout the summer, under the influence of natural variations of climatic conditions. We fully avoided water and nutrients stress throughout plants’ development by applying daily irrigation as it was needed. Details of germination protocol, acclimation, soil physical and chemical profile, as well as growth conditions are described in **Supporting Information A**.

### Air temperature thresholds, heat sum in degrees days and phenology

The daily minimum and maximum air temperature thresholds were recorded in the different sunlight regimes and open sky **[see****Supporting Information A****]**. The heat sum in degrees days (*°*D) was quantified throughout plants’ phenological development (Moreno *et al.* 2014). Plants’ phenological transition is shown in Table 2.

**Table 1.** Standard symbols and abbreviations used in the text, their units and definitions. Some symbols or abbreviations that appear alongside their definitions in the text were not included here.

Symbol	Units	Definition
PPFD	µmol m^−2^ s^−1^	Photosynthetically active photons flux density
VPD	kPa	Vapour pressure deficit
CO_2_	ppm	Carbon dioxide
*T*_leaves_	°C	Temperature of leaf at the time of gas exchange measurements
*C*_3_	—	The most common metabolic pathway for carbon fixation through photosynthesis
*R*_dark_	µmol CO_2_ m^−2^ s^−1^	Respiration rate in the dark, i.e. in absence of light
LCP	µmol CO_2_ m^−2^ s^−1^	Light compensation point
PPFD_50_	µmol m^−2^ s^−1^	Photosynthetically active photons flux density which half saturates CO_2_ assimilation
PPFD_95_	µmol m^−2^ s^−1^	Photosynthetically active photons flux density which saturates CO_2_ assimilation by 95 %
*g*_s_	mmol m^−2^ s^−1^	Stomatal conductance
*T*_r_	mmol H_2_O m^−2^ s^−1^	Transpiration rate
*A/g*_s_	µmol CO_2_ mmol H_2_O	Intrinsic photosynthetic water use efficiency or the ratio between *A/g*_s_
*A/T*_r_	µmol CO_2_ mmol H_2_O	Instantaneous photosynthetic water use efficiency or the ratio between *A/T*_r_
*g*_m_	µmol CO_2_ m^−2^ s^−1^ Pa^−1^	Mesophyll conductance
*C*_a_	µmol mol^−1^	Ambient CO_2_ concentration
*C*_i_	µmol CO_2_ mol^−1^ or Pa	Intercellular CO_2_ concentration or the CO_2_ partial pressure at intercellular spaces
*C*_c_	µmol CO_2_ mol^−1^ or Pa	Chloroplastic CO_2_ concentration or the CO_2_ partial pressure at the carboxylation sites of Rubisco
*l*_s_, *l*_m_, *l*_b_	%	Stomatal, mesophyll and biochemical limitations of photosynthesis, respectively
*A*	µmol CO_2_ m^−2^ s^−1^	net rate of CO_2_ assimilation
*R*_d_ */A*	µmol CO_2_/µmol CO_2_ m^−2^ s^−1^	The ratio between CO_2_ respiration and CO_2_ assimilation
*A*_max_	µmol CO_2_ m^−2^ s^−1^	The maximum CO_2_ assimilation at high concentrations of CO_2_ or at high levels of irradiance
Rubisco	—	Ribulose 1–5 bisphosphate carboxilase-oxigenase
Rubp	—	Ribulose 1–5 bisphosphate
*Ac*	—	The portion of the photosynthetic process limited by the Rubisco activity
*Aj*	—	The portion of the photosynthetic process limited by the Rubp regeneration
*C*_ctr_	Pa	The transitory portion between *Ac* and *Aj* evaluated at *C*_c_
*J*/4	—	Four electrons are required for every assimilated CO_2_ and every O_2_ evolved
*V*_cmax_	µmol CO_2_ m^−2^ s^−1^	Maximum velocity of Rubisco carboxylation
*J*_max_	µmol e^−^ m^−2^ s^−1^	Maximum electrons transport rate
*J*	µmol e^−^ m^−2^ s^−1^	Electrons transport rate
TPU	µmol CO_2_ m^−2^ s^−1^	Triose phosphate utilization rate
*R*_d_	µmol CO_2_ m^−2^ s^−1^	Respiration rate in the day, i.e. in presence of light
Г*	µmol mol^−1^	Chloroplastic CO_2_ photocompensation point or the CO_2_ required to overcome photorespiration
Г	µmol mol^−1^	CO_2_ compensation point or the CO_2_ required to overcome *R*_d_ or where *A* = 0

**Table 2.** Degrees days (°D) throughout phenological stages of *Capsicum annuum* var. *glabriusculum* plants grown in high, intermediate and low sunlight regimes. Survival rate after 157 days of growth is also shown (*n* = 36 plants per treatment). The chronological timescale in days, the day of year, months and seasons are shown. *Indicates the day of plants transplantation.

Phenological stages	Sowing time	Seedling birth	*Early vegetative growth	Early flowering	Full vegetative or reproductive growth	Survival rate %	Flowers and Fruits
Treatments	(°D) Degrees days throughout phenological stages						
High sunlight regimes	0	521	1042	1401	4035	5.5	No
Intermediate sunlight regimes	0	516	1032	1397	3857	86.1	Yes
Low sunlight regimes	0	482	964	1284	3441	91.6	No
Timescale in days	0	15	49	63	157		
Day of year	108	123	157	171	265		
Months	April, May, June			June, July, August, September			
Season	Spring			Summer			

### Survival rate and gas exchange measurements

Three days before gas exchange measurements, the survival rate (%) of plants was quantified at the stage of full vegetative and reproductive growth. Although there was no water and nutrients stress, in high sunlight regimes very few plants survived (Table 2). Therefore, all measurements were conducted only in plants grown in low and intermediate sunlight. The gas exchange measurements were conducted at the stage of full vegetative and reproductive growth in summer (Table 2); CO_2_ assimilation curves in response to the photosynthetic photon flux density (*A*/PPFD) and CO_2_ concentrations (*A/C*_i_) were measured. Before this study, we conducted gas exchange measurements in wild plants to get reference values to compare those traits of cultivated plants. It was used a single portable infrared gas analyser system following standard procedures (LICOR 6400XT™). For details of gas exchange measurements, see **Supporting Information A**, while the raw gas exchange data set in **Supporting Information B**.

### Curves fitting, parameters validation and biochemical traits assessment

Definitions and units from photosynthetic parameters are shown in Table 1. The measured *A*/PPFD curves and its photosynthetic parameters (i.e. *A*_max_, *R*_dark_, *J*_max_, others) were solved (i.e. validated) using different methods (Lobo *et al.* 2013; Sharkey 2016; www.landflux.org). The measured *A/C*_i_ curves were corrected to *A*/*C*_c_ curves by using the respective equation, removing by this way all diffusion resistance effects. Afterward, the *A*/*C*_c_ curves were solved by using two different curve fitting methods, e.g. the linear and rectangular mathematical methods (Sun *et al.* 2014; Sharkey 2016, 2019). From the *A/C*_c_ curves, multiple photosynthetic parameters were derived either directly or by using the corresponding equations (i.e. *V*_cmax_, *A*_max_, *R*_d_*/A*, *C*_ctr_, others) (Martins *et al.* 2014; Sun *et al.* 2014; Sharkey 2016; www.landflux.org). To identify the photosynthetic plasticity traits, the gas exchange parameters recorded on wild plants were compared to cultivated plants in low and intermediate sunlight. A detailed description of curve fitting, and parameters validation is shown in **Supporting Information A**. Immediately after gas exchange measurements, leaves were freezing (−20°C) until analysis of total photosynthetic pigments, chlorophyll a, chlorophyll b, xanthophylls and carotenoids (Lichtenthaler and Buschmann 2001). All canopy leaves from harvested plants were dried, grounded to a fine powder and then subsamples were used to quantify the macroelements and microelements (FLASH 2000 analyser™, Thermo Scientific™; UV–visible spectrophotometer). Afterward, the total content of each macroelement and microelement was calculated on a leaf area basis. To calculate the amount of nitrogen allocated to the main protein complexes of photosynthetic machinery (i.e. carboxylation, bioenergetics and light-harvesting), we applied the approach, equations and constants proposed by Niinemets and Tenhunen (1997). The leaf anatomical traits related to the gas exchange capacity were evaluated under the optical microscope (Leica BX51) by counting the abaxial and adaxial stomatal density, as well as the abaxial stomatal index (Martins *et al.* 2014).

### Plants harvest, growth rate, biomass allometry and elemental analysis

Immediately after gas exchange measurements, plants harvest for growth assessment was conducted, six plants from the low sunlight and six plants from the intermediate sunlight. We quantified plants’ growth rate, architectural development and their biomass allometry through different parameters: (i) relative growth rates, (ii) leaf area index, (iii) individual leaf area, (iv) specific leaf area, (v) leaf area ratio, (vi) total dry biomass, (vii) leaf mass per area, (viii) shoot/roots ratio, (ix) roots mass fraction, (x) stems mass fraction, (xi) leaf mass fraction and (xii) specific stem length (Poorter *et al.* 2012c, 2015). The reproductive yield was quantified by measuring the fruits’ fresh and dry weight, and the reproductive mass fraction on a dry basis.

### Experimental design and statistical analysis

All statistical tests were conducted in NCSS 2007. Before statistical analysis, the Skewness and Kurtosis tests were used to check normality, as well as to evaluate the variance homogeneity, Levene’s test was applied. A one-way analysis of variance and a Fisher LSD test (*P* ˂ 0.05) were performed with the photosynthetic parameters derived with different curve fitting methods. Reference gas exchange parameters from wild plants (*n* = 4) were compared to those derived from cultivated plants (*n* = 6). Simultaneously, gas exchange parameters from plants grown in low sunlight (*n* = 6) were compared to those of plants grown under intermediate sunlight (*n* = 6). Likewise, the architectural, anatomical, biochemical and growth traits from cultivated plants (*n* = 6) were analysed. We conducted a multiple correlation analysis by using the significant traits **[see****Supporting Information B****]**. We also applied a principal components analysis through the varimax method, by using the raw data of multiple traits to extract those most meaningful from the statistical point of view **[see****Supporting Information B****]**.

## Results

### Climatological trend and phenological response throughout the ecophysiological timeline

**Figure 1.**
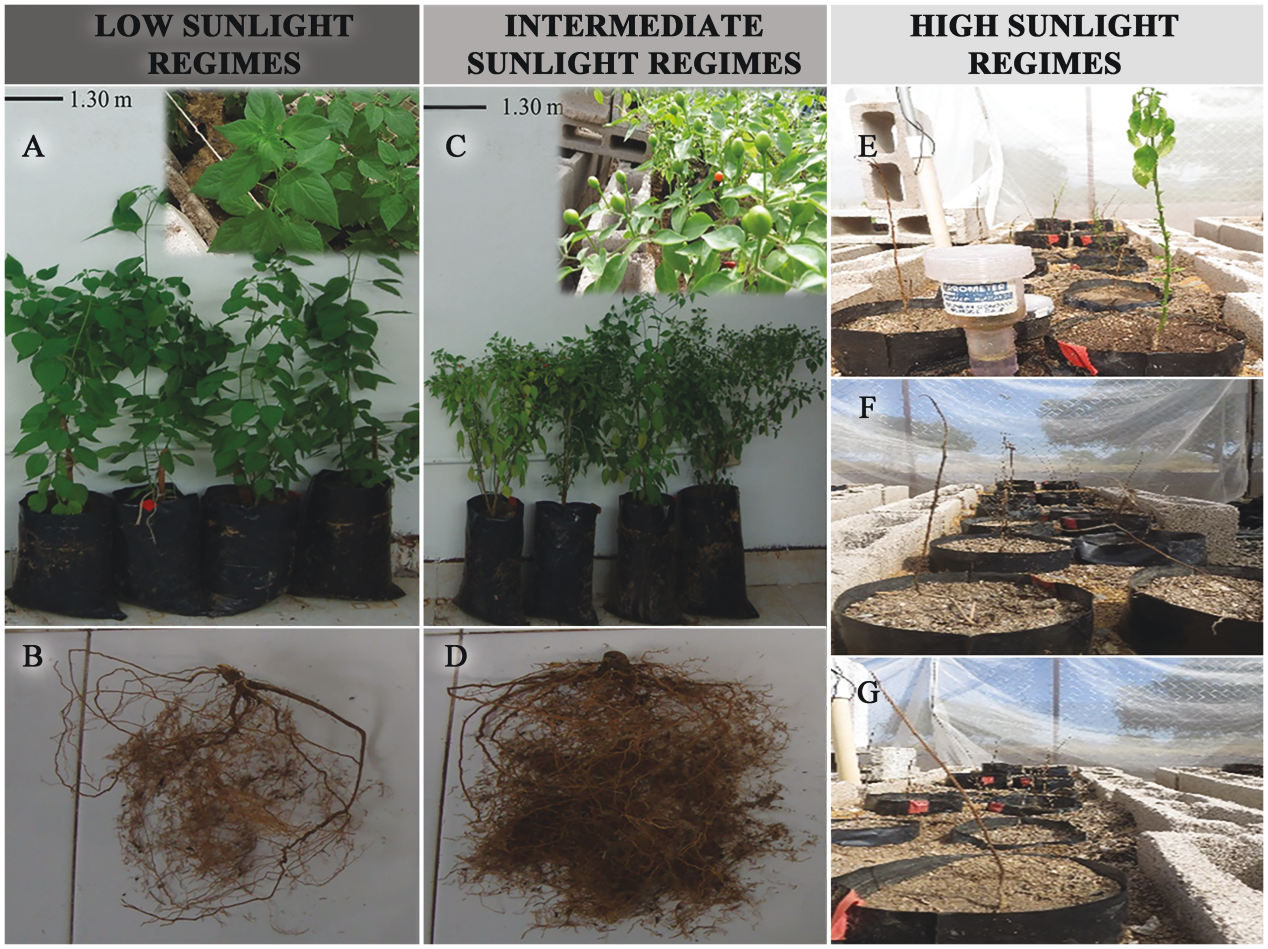
*Capsicum annuum* var*. glabriusculum* plants harvested in low (A), and intermediate (C) sunlight regimes, after 157 days of development. In the high sunlight regimes (E–G) there was no harvest since most plants died. In (A) vegetative growth (stems and leaves) and (B) a sample of roots. In (C) the vegetative and reproductive growth (stems, leaves and green fruits), and (D) a sample of roots.

At open sky the climatological conditions (i.e. sunlight regimes, air temperatures, rainfalls and relative humidity) were contrasting throughout the experiment during spring and summer **[see****Supporting Information A—Fig. S2****]**. Under the different shading nets, plants developed under contrasting sunlight regimes and extremely warm air temperature thresholds **[see****Supporting Information A—Table S2****]**; therefore, a high heat sum was rapidly accumulated throughout plants phenological stages from seeds sowing until the full vegetative and reproductive growth (Table 2). The daily maximum air temperature thresholds recorded under the different sunlight regimes were significantly higher than the daily maximum air temperature thresholds recorded at the open sky. During the summer, the extremely warm air temperature thresholds recorded at the different sunlight regimes promoted warm soil temperatures **[see****Supporting Information A—Table S3****]**. Plants showed a contrasting phenological response and survival rate. Under the high sunlight regimes, there were not flowers and fruits production since most plants did not develop, but on the contrary almost all died, i.e. there was only 5 % of survival in this treatment (Table 2; Fig. 1). By contrast, under intermediate sunlight regimes plants developed vegetatively, the survival rate was high (86 %) and production of flowers and fruits was positive. In the low sunlight regimes plants developed vegetatively, the survival rate was high (91 %), but there were not flowers and fruits production (Table 2; Fig. 1).

### Curves validation and photosynthetic traits

**Figure 2.**
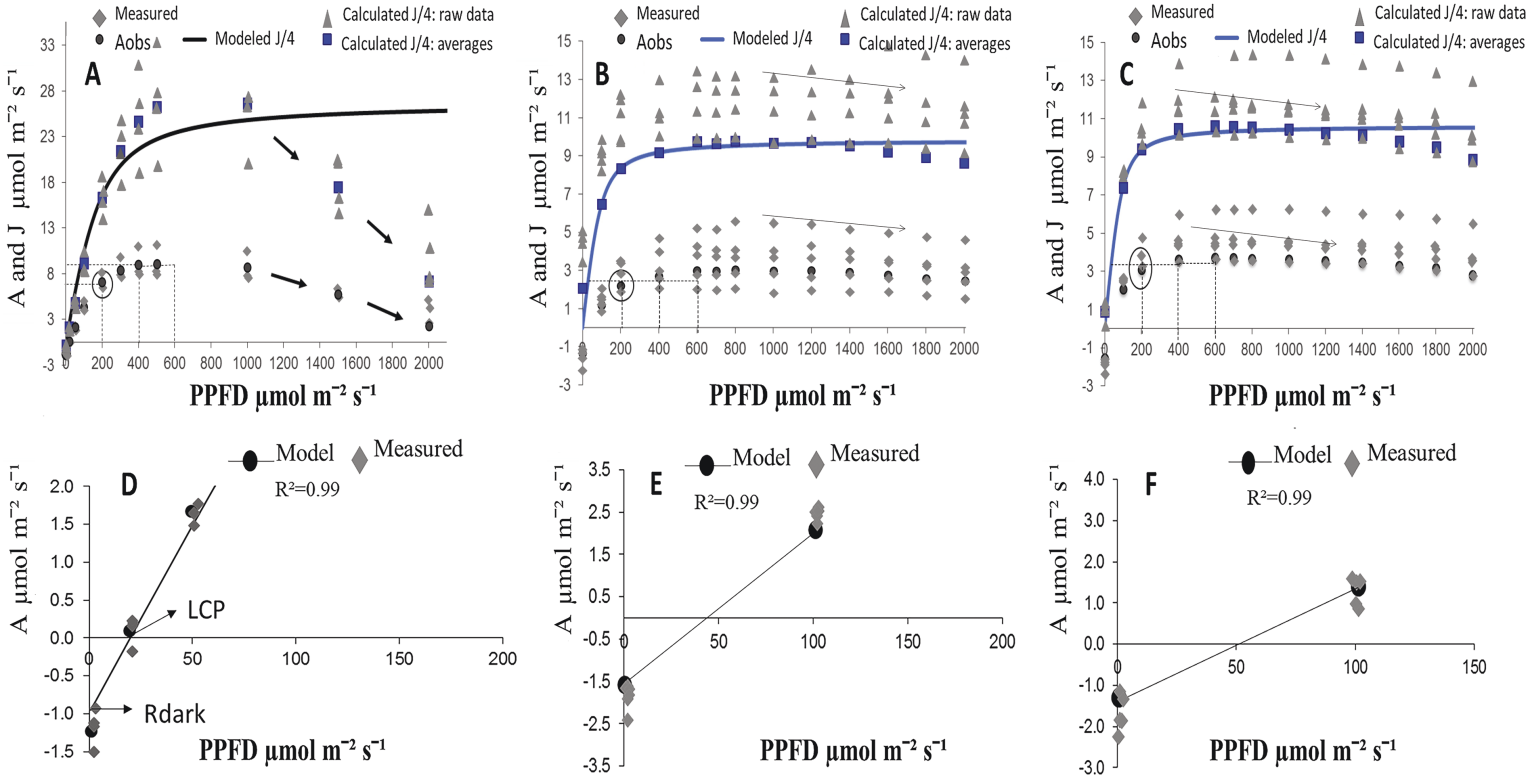
CO_2_ assimilation curves in response to photosynthetic photons flux density (i.e. *A*-PPFD) measured on wild plants (A, D, *n* = 4) and cultivated plants in low and intermediate sunlight regimes (B, C, E, F, *n* = 6). In (A–C): 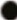 Aobs refers to the averages from measured points. 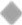 refers to measured data. 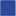 refers to averages from calculated *J*/4. 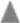 refers to the raw data from calculated *J*/4. In (A–C): the continuous line refers to the modelled *J*/4. In (A–C): the curvature point and saturation of curves are shown. In (A–C): arrows indicate the declination of *A* at high PPFD. The LCP and *R*_dark_ are shown in the linear portion of curves (D–F). The measured points and those predicted by the model are shown in the linear portion of the curves (D–F).

**Figure 3.**
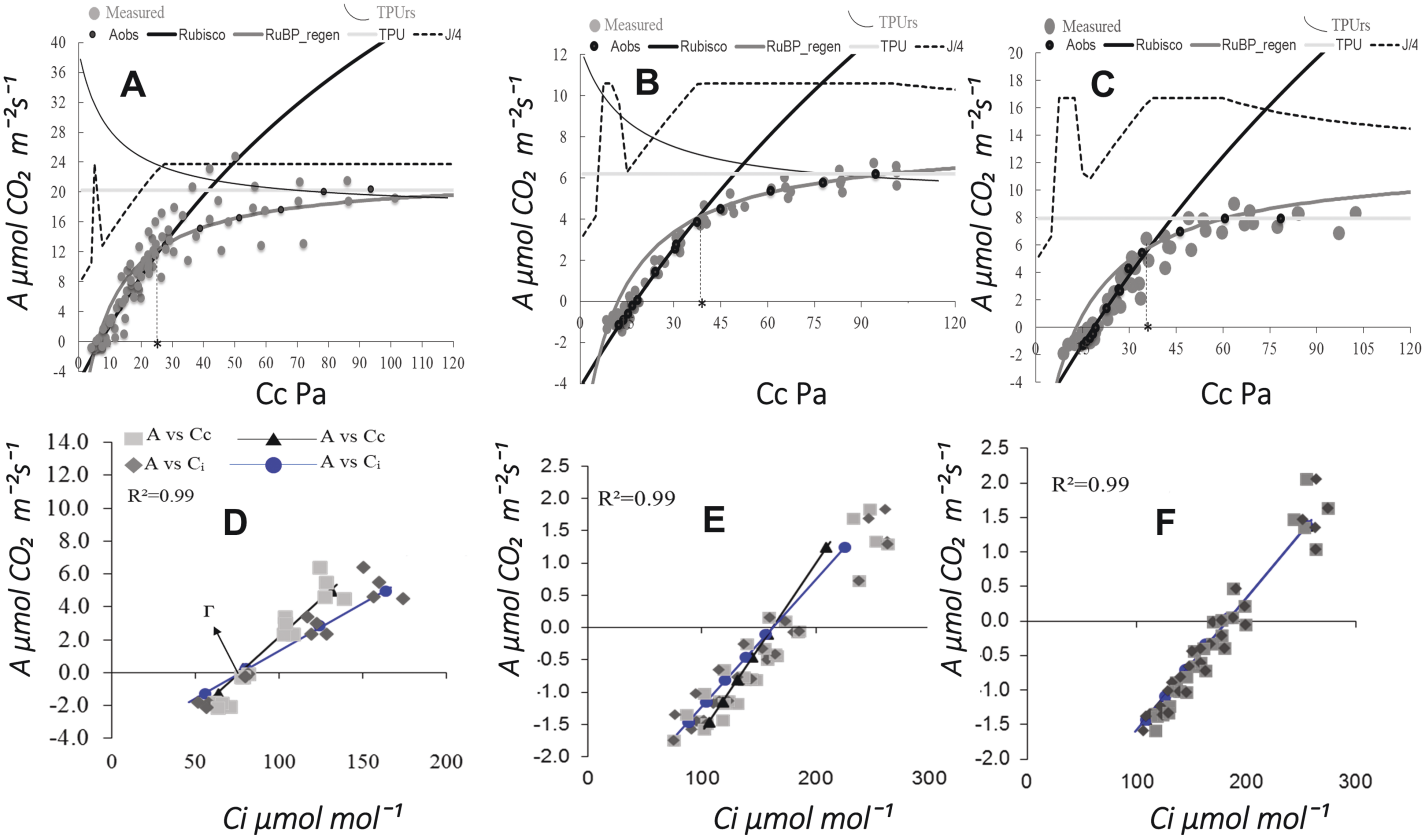
(A, C) Photosynthetic assimilation curves in response to chloroplastic CO_2_ partial pressures (*A*/*C*_c_); and the intercellular CO_2_ concentration (*A*/*C*_i_) in the lineal portion (D, F). Curves were measured on wild plants (A, D, *n* = 4), and cultivated plants in low and intermediate sunlight regimes (B, C, E, F, *n* = 6). The measured points and those predicted by model are shown (A, F). The Rubisco, Rubp, TPU, TPUrs and *J*/4 are shown (A, C). Aobs, average of observed points with respect of measured points (A, C), * Ciopt = indicates that *Ac* = *Aj* (A, C). The CO_2_ compensation point (Г) is shown in the linear portion (D, F). In the linear portion (D, F): 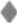
*A/C*_i_ curves. 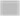
*A/C*_c_ curves. 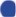 Averages from the *A*/*C*_i_ curves. 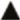 Averages from the *A/C*_c_ curves. Sum of square errors ˂1.

The measured data from the photosynthetic curves in response to light and CO_2_ (i.e. *A*/PPFD, *A*/*C*_i_ curves) (Figs. 2 and 3) fitted mathematically to data predicted by the biophysical and biochemical model of photosynthesis. In the case of photosynthetic curves in response CO_2_ (Fig. 3), when two different mathematic fitting methods were applied, the derived photosynthetic parameters were similar **[see****Supporting Information A—Table S5****]**. The photosynthetic curves in response to light (Fig. 2) showed different traits at different conditions. The curves initial slope and the curvature were similar in wild and cultivated plants (Table 3; **see****Supporting Information A— Table S4**). As compared to cultivated plants, wild plants showed higher maximum CO_2_ assimilation (*A*_max_), and maximum electrons transport efficiency (*J*_max_, *J*/4) (Fig. 2; Table 3). The dark respiration (*R*_dark_) was similar in wild and cultivated plants under the intermediate sunlight regimes, but respiration tended to be higher in plants grown in low sunlight regimes (Table 3). The light compensation point (LCP) from wild versus cultivated plants was lower. On average, the CO_2_ assimilation reached the 50 % of light saturation between 80 and 111 μmol m^−2^ s^−1^ of irradiance (PPFD_50_), it reached 95 % light saturation between 256 and 491 μmol m^−2^ s^−1^ (PPFD_95_), it showed the asymptotic trend between 500 and 1000 μmol m^−2^ s^−1^ and its declination at irradiance levels between 1200 and 1400 μmol m^−2^ s^−1^ (Fig. 2; Table 3). The derived parameters from the photosynthetic curves in response to light (Fig. 2) and the photosynthetic curves in response to CO_2_ (Fig. 3) reflected the maximum functional capacity of plants in summer just at the stage of full growth (Table 2). In addition, multiple photosynthetic acclimation traits were recorded (Table 4).

**Table 3.** Photosynthetic traits (A/PPFD) measured on leaves from wild and cultivated *Capsicum annuum* var. *glabriusculum* plants. Average contrast is by column. Averages with different letters were significant *P* ˂ 0.05. SSE: sum of square error. *: Buckley and Diaz-Espejo (2015) and Sharkey (2016).

Plants	Photosynthetic traits											
	*T*_leaf_ (°C)	VPD (kPa)	RH (%)	*R*_dark_ (µmol m^−2^ s^−1^)	LCP (µmol m^−2^ s^−1^)	Initial slope (ɸ)	PPFD_50_ (µmol m^−2^ s^−1^)	Curvature	PPFD_95_ (µmol m^−2^ s^−1^)	*A*_max_ (µmol CO_2_ m^−2^ s^−1^)	*J*_max_ (µmol e^−^ m^−2^ s^−1^)	SSE
Wild plants	34 ± 0.9ª	4 ± 0.4ª	25 ± 5ª	1.2 ± 0.09ª	19 ± 2ª	0.39 ± 0.04ª	111 ± 8ª	0.8 ± 0.04ª	491 ± 97ª	9.8 ± 1ª	*J_1000_ 101 ± 18ª	0.6 ± 0.3
Cultivated plants in low sunlight	36 ± 1ᵇ	2 ± 0.4ᵇ	64 ± 4ᵇ	1.8 ± 0.2ᵇ	40 ± 3ᵇ	0.43 ± 0.06ª	80 ± 18ªᵇ	0.75 ± 0.1ª	256 ± 68ᵇ	6 ± 1ᵇ	*J_1400_ 46 ± 9ᵇ	0.9 ± 0.1
Cultivated plants under intermediate sunlight	40 ± 2^c^	2 ± 0.7ᵇ	65 ± 7ᵇ	1.4 ± 0.3ªᵇ	48 ± 12ᵇ	0.36 ± 0.1ª	99 ± 51ª	0.82 ± 0.1ª	303 ± 176ᵇ	5 ± 1ᵇ	*J_1400_ 50 ± 33ᵇ	0.8 ± 0.5

**Table 4.** Photosynthetic traits of leaves from wild and cultivated *Capsicum annuum* var. *glabriusculum* plants. Average contrast is by row. Averages with the different letters were significant *P* ˂ 0.05. * indicates that the highest average was recorded in leaves from wild plants.

Photosynthetic traits	Wild plants	Cultivated plants	
		Treatments	
		Low sunlight	Intermediate sunlight
Air temperature (°C)	33 ± 1^a^	40 ± 2^b^	40 ± 5^b^
Irradiance (PPFD = µmol e^−^ m^−2^ s^−1^)	399 ± 0.6^a^	95 ± 0.6^b^	652 ± 8^c^
Leaf temperature (°C)	33 ± 1^a^	39 ± 2^b^	39 ± 4^b^
Vapour pressure deficit (VPD = kPa)	2.9 ± 0.4^a^	3.0 ± 0.8^a^	1.7 ± 0.8^b^
*g*_s_ (mmol H_2_O m^−2^ s^−1^)	122 ± 55^a^	215 ± 73^a^	384 ± 158^b^
*T*_r_ (mmol H_2_O m^−2^ s^−1^)	3.6 ± 1^a^	6 ± 1^b^	5.7 ± 2.2^ab^
*A/g*_s_ ratio (µmol CO_2_ mmol H_2_O)	*0.072 ± 0.01^a^	0.013 ± 0.002^b^	0.0081 ± 0.003^c^
*A/T*_r_ ratio (µmol CO_2_ mmol H_2_O)	*2.2 ± 0.5^a^	0.47 ± 0.1^b^	0.59 ± 0.3^b^
*g*_m_ (µmol CO_2_ m^−2^ s^−1^)	0.12 ± 0.05^a^	0.12 ± 0.07^a^	0.045 ± 0.03^b^
*C*ᵢ (µmol CO_2_ mol^−1^ air)	251 ± 28^a^	351 ± 7^b^	367 ± 8^c^
*C*_c_ (µmol CO_2_ mol^−1^ air)	176 ± 19^a^	321 ± 16^b^	289 ± 34^c^
*g*_m_/*g*_s_ ratio (µmol CO_2_ mol^−1^ CO_2_)	*1.1 ± 0.6^a^	0.60 ± 0.4^a^	0.12 ± 0.07^b^
*l*_s_ * 100 = %	*0.87 ± 0.05^a^	0.61 ± 0.07^b^	0.45 ± 0.9^c^
*l*_m_ * 100 = %	0.06 ± 0.03^a^	0.081 ± 0.03^a^	0.26 ± 0.08^b^
*l*_b_ * 100 = %	0.06 ± 0.02^a^	0.30 ± 0.08^b^	0.27 ± 0.12^b^
*A*_max_ (µmol CO_2_ m^−2^ s^−1^)	*17.6 ± 3^a^	6.2 ± 0.4^b^	8 ± 0.6^c^
*A* (µmol CO_2_ m^−2^ s^−1^)	*8.2 ± 1.7^a^	2.8 ± 0.4^b^	2.8 ± 0.7^b^
*R*_d_/*A* ratio (µmol CO_2_/µmol CO_2_ m^−2^ s^−1^)	0.2 ± 0.1^a^	0.9 ± 0.2^b^	1.6 ± 0.8^c^
*V*_cmax_ (µmol CO_2_ m^−2^ s^−1^)	102 ± 39^a^	52 ± 10^b^	92 ± 34^a^
*J* (µmol e^−^ m^−2^ s^−1^)	*93 ± 17^a^	41 ± 2^b^	65 ± 12^c^
*J/V*_cmax_ ratio	0.95 ± 0.1^a^	0.83 ± 0.2^a^	0.80 ± 0.1^a^
*C*_ctr_ (Pa)	23 ± 4^a^	39 ± 4^b^	39 ± 9^b^
TPU (µmol CO_2_ m^−2^ s^−1^)	*6.4 ± 1^a^	2.9 ± 0.1^b^	4.2 ± 0.6^c^
*R*_d_ (µmol CO_2_ m^−2^ s^−1^)	1.5 ± 0.4^a^	2.5 ± 0.2^b^	4.9 ± 1^c^
Г (µmol CO_2_ mol^−1^ air)	63 ± 5^a^	164 ± 13^b^	174 ± 27^b^
Г* (µmol CO_2_ mol^−1^ air)	54 ± 2^a^	76 ± 6^b^	74 ± 12^b^

Contrasting traits were recorded from the wild versus cultivated plants (Table 4; **see****Supporting Information A—Table S6**). First, as compared to wild plants, in the low and intermediate sunlight regimes, the cultivated plants photosynthetically responded to warmer temperatures, different irradiance and different vapour pressure deficit. Some traits were higher in wild versus cultivated plants, e.g. photosynthetic capacity (*A*_max_, *A*, *J*, triose phosphate utilization [TPU]), the photosynthetic water use efficiency (*A/g*_s_, *A/T*_r_), the ratio between mesophyll and stomatal conductance (*g*_m_/*g*_s_ ratio), as well as the stomatal limitation (*l*_s_). However, other specific traits were lower in wild versus cultivated plants, e.g. the ratio between CO_2_ respiration and CO_2_ assimilation (*R*_d_/*A*), respiration (*R*_d_), CO_2_ compensation and photocompensation point (Г, Г*), as well as the proportional mesophyll and biochemical limitations (*l*_m_, *l*_b_). In wild plants, the mean CO_2_ concentration drawdown from intercellular spaces (*C*_i_) towards chloroplastic spaces (*C*_c_) (74 µmol CO_2_ mol^−1^) was lower than those from ambient (*C*_a_) towards intercellular spaces (*C*_i_) (148 µmol CO_2_ mol^−1^) (Table 4). In plants grown under intermediate sunlight, the mean CO_2_ concentration drawdown from intercellular spaces towards chloroplastic spaces (78 µmol CO_2_ mol^−1^) was higher than those from ambient towards intercellular spaces (32 µmol CO_2_ mol^−1^). Similarly, in leaves plants grown in low sunlight, the mean CO_2_ concentration drawdown from intercellular spaces towards chloroplastic spaces (29 µmol CO_2_ mol^−1^) was lower than those from ambient towards intercellular spaces (48 µmol CO_2_ mol^−1^) (Table 4). In response to low and intermediate sunlight regimes, cultivated plants exhibited contrasting traits, e.g. the CO_2_ and water diffusional efficiency (*g*_s_, *T*_r_, *A/g*_s_, *g*_m_/*g*_s_ ratio), photosynthetic capacity traits (*A*_max_, *R*_d_/*A* ratio, *V*_cmax_) and proportional limitation in stomata and mesophyll (*l*_s_, *l*_m_). However, other specific photosynthetic traits were similar in cultivated plants (*T*_r_, *A/T*_r_, *l*_b_, *A*, *J/V*_cmax_, *C*_ctr_, Г, Г*).

### Architectural, anatomical and biochemical traits

The specific and individual leaf area, and the leaf area ratio were higher in plants grown in low sunlight regimes (Table 5; **see****Supporting Information A—Table S7**). The leaf mass per area, adaxial and abaxial stomatal density, as well as the stomatal index were higher in plants grown under intermediate sunlight regimes, which also increased their photosynthetic pigments, nitrogen allocation to photosynthetic components and the content of macroelements and microelements (e.g. C, H, S, P, Mg, Ca, Fe, Ni, Cu). However, the content of some specific elements was similar in plants grown under the different sunlight regimes (e.g. N, K, Na, Zn and Mn).

**Table 5.** Architectural, anatomical and biochemical traits from *Capsicum annuum* var. *glabriusculum* plants grown under different sunlight regimes. Average contrast is by row. Averages with the different letters were significant *P* ˂ 0.05.

Traits	Treatments	
	Low sunlight regimes	Intermediate sunlight regimes
Specific leaf area (SLA = m^2^ g^−1^)	0.07 ± 0.01^a^	0.03 ± 0.004^b^
Leaf area ratio (LA R= m^2^ g^−1^)	0.02 ± 0.004^a^	0.005 ± 0.001^b^
Individual leaf area (ILA = cm^2^)	18 ± 4^a^	4.6 ± 1^b^
Leaf mass per area (LMA = g m^−2^)	13 ± 1^a^	32 ± 5^b^
Adaxial stomatal density per cm^2^	0	127 ± 0.2
Abaxial stomatal density per cm^2^	191 ± 52^a^	438 ± 57^b^
Stomatal index (%)	12 ± 4^a^	25 ± 1^b^
Total photosynthetic pigments (TPP = g m^−2^)	0.4 ± 0.05^a^	1.5 ± 0.1^b^
Chlorophyll a (*Chla* = mg m^−2^)	277 ± 33^a^	770 ± 102^b^
Chlorophyll b (*Chlb* = mg m^−2^)	127 ± 14^a^	501 ± 91^b^
Xanthophylls plus carotenoids (mg m^−2^)	72 ± 9^a^	237 ± 45^b^
Nitrogen (N = g m^−2^)	0.51 ± 0.1^a^	0.66 ± 0.1^a^
Nitrogen to carboxylation (NC = mg g N m^−2^)	118 ± 7^a^	187 ± 58^b^
Nitrogen to bioenergetics (NB = mg g N m^−2^)	14 ± 1^a^	22 ± 4^b^
Nitrogen to light-harvesting (NL = mg g N m^−2^)	35 ± 9^a^	133 ± 23^b^
Nitrogen to photosynthetic components (NP = mg g N m^−2^)	169 ± 14^a^	343 ± 62^b^
NL/NP (mg g^−1^)	0.20 ± 0.04^a^	0.39 ± 0.09^b^
Carbon (g m^−2^)	5.9 ± 0.9^a^	14 ± 2^b^
Sulphur (S = g m^−2^)	0.07 ± 0.02^a^	0.23 ± 0.07^b^
Hydrogen (H = g m^−2^)	0.8 ± 0.1^a^	1.9 ± 0.2^b^
Phosphorus (P = g m^−2^)	0.05 ± 0.02^a^	0.13 ± 0.02^b^
Magnesium (Mg = g m^−2^)	0.06 ± 0.01^a^	0.18 ± 0.04^b^
Calcium (Ca = g m^−2^)	0.19 ± 0.03^a^	0.34 ± 0.1^b^
Potasium (K = g m^−2^)	0.04 ± 0.01^a^	0.04 ± 0.01^a^
Sodium (Na = g m^−2^)	0.09 ± 0.03^a^	0.1 ± 0.02^a^
Manganese (Mn = mg m^−2^)	1.3 ± 0.3^a^	1.5 ± 0.2^a^
Iron (Fe = mg m^−2^)	6.4 ± 1.5^a^	8.7 ± 0.9^b^
Nickel (Ni = mg m^−2^)	0.03 ± 0.02^a^	0.14 ± 0.02^b^
Zinc (Zn = mg m^−2^)	0.6 ± 0.2^a^	0.9 ± 0.4^a^
Copper (Cu = mg m^−2^)	0.2 ± 0.07^a^	0.51 ± 0.1^b^

### Growth rate and biomass allometry

The relative growth rate of plants grown under intermediate sunlight regimes was higher; consequently, their total biomass, fruits fresh and dry weight, roots and reproductive mass fraction were higher. By contrast, the ratio of shoots and roots, leaf mass fraction and specific stems length were higher in plants grown in low sunlight regimes. The leaf area index and stems mass fraction were similar (Table 6; **see****Supporting Information A—Table S8**).

**Table 6.** Growth rate and biomass allometry of *Capsicum annuum* var. *glabriusculum* plants grown in different sunlight regimes. Average contrast is by row. Averages (*n* = 6) with different letters were significant *P* ˂ 0.05.

Growth and biomass allometry	Treatments	
	Low sunlight	Intermediate sunlight
Relative growth rate (RGR = g g^−1^ day^−1^)	0.04 ± 0.03^a^	0.19 ± 0.1^b^
Total biomass (TB = g)	4.9 ± 2.8^a^	21 ± 13^b^
Leaf area index (LAI = m^2^ m^−2^)	0.13 ± 0.06^a^	0.10 ± 0.06^a^
Fruits fresh weight (FFW = g)	0	5.5 ± 3
Fruits dry weight (FDW = g)	0	2.02 ± 1
Shoots/roots ratio (S/R ratio = g g^−1^)	3.8 ± 0.8^a^	2 ± 0.3^b^
Roots mass fraction (RMF = g g^−1^)	0.21 ± 0.03^a^	0.3 ± 0.03^b^
Stems mass fraction (SMF = g g^−1^)	0.41 ± 0.07^a^	0.39 ± 0.07^a^
Leaves mass fraction (LMF = g g^−1^)	0.37 ± 0.06^a^	0.15 ± 0.02^b^
Reproductive mass fraction (REMF = g g^−1^)	0	0.14 ± 0.1
Specific stem length (SSL = cm g^−1^)	36 ± 14^a^	6 ± 4^b^

### Principal components analysis

**Figure 4.**
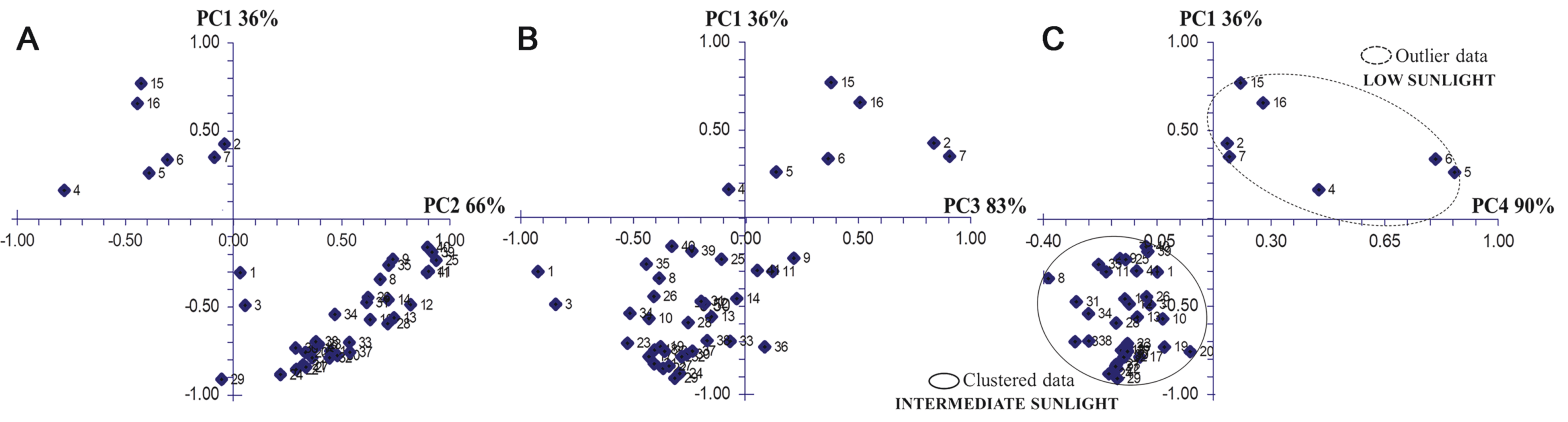
Principal components and their accumulated eigenvalues. Points in graphs represent the average of each evaluated traits and its loadings towards the principal components. The outlier and clustered data are shown (NCSS v2007). Outlier data = 2: *A*/*g*_s_ ratio, 4: *C*_c_, 5: *g*_m_, 6: *g*_m_*/g*_s_ ratio, 7: *l*_s_, 15: SLA, 16: LAR. Clustered data = 1: *g*_s_, 3: *C*_i_, 8: *l*_m_, 9: *R*_d_/*A* ratio, 10: *A*_max_, 11: *V*_cmax_, 12: *J*, 13: TPU, 14: *R*_d_, 17: LMA, 18: SD_ad_, 19: SD_ab_, 20: SI_ab_, 21: TPP, 22: *Chla*, 23: *Chlb*, 24: Xant–Carot, 25: NC, 26: NB, 27: NL, 28: NL/NP, 29: C, 30: S, 31: H, 32: P, 33: Mg, 34: Ca, 35: Fe, 36: Ni, 37: Cu, 38: RGR, 39: TB, 40: REMF. Definitions of all abbreviations are shown in Table 1 and **Supporting Information B**.

The principal components analysis through the varimax method showed that the accumulated eigenvalues from four principal components explained 90 % of the total experimental variance (PC1 = 35 %, PC2 = 31.7 %, PC3 = 16.3 % and PC4 = 6.8 %) (Fig. 4A and C). After the varimax rotations around four principal components, the resulting data clouds around the principal components were distributed as follows: A majority set (34 in total) of clustered data corresponding to significant traits of plants grown under intermediate sunlight regimes and a minority set (7 in total) of outlier data dispersed towards the opposite side of clustered data corresponding to significant traits of plants grown in low sunlight regimes (Fig. 4A and C). Towards its extreme top, PC1 (Fig. 4A) correlated to the specific leaf area and towards its extreme bottom to the ratio between nitrogen allocation to light-harvesting and photosynthetic components. Towards its extreme right, PC2 (Fig. 4A) correlated to the nitrogen allocation to carboxylation, and towards its extreme left to chloroplastic CO_2_ concentration. Towards its extreme right, PC3 (Fig. 4B) correlated to the stomatal limitation and towards its extreme left to stomatal conductance. Towards its extreme right, PC4 (Fig. 4C) correlated to the mesophyll conductance and towards its extreme left to mesophyll limitation.

## Discussion

### Climatological trend, survival rate and phenological response

The climatological record during plants development is fundamental in phenological and ecophysiological terms (Wadgymar *et al.* 2018; Poorter *et al.* 2019). In this study, the extreme climatological conditions at open sky **[see****Supporting Information A—Fig. S2****]** reflected the typical seasonal trend of the Sonoran Desert (CONAGUA 2014). In a window of 157 days of growth, the sum of heat in degrees days was extremely high in the low, intermediate and high sunlight regimes (Table 2). Such extreme heating under the shading nets was promoted by the warm air temperature thresholds recorded at open sky throughout spring and summer. During summer, the extremely warm temperatures plants tolerated under the shading nets were higher than temperatures recorded in the open sky (11–38 %). It can be attributed to a heat-trapping effect caused by the shading nets (Pérez *et al.* 2006). Jiménez-Leyva *et al.* (2017) successfully cultivated the same species under intermediate sunlight regimes and extremely warm temperature thresholds with a fast accumulation of heat in degrees days.

Although plants developed without hydric and nutritional stress, their phenological response and survival were contrasting. The high sunlight regimes and extremely warm temperature thresholds promoted a fast detrimental effect for plants because they induced growth arrest, progressive yellowing and defoliation until eventually most plants died (Fig. 1; Table 2). By contrast, plants grown under the intermediate sunlight regimes and extremely warm temperatures showed a fast vegetative and reproductive growth with high survival. Unexpectedly, plants grown in low sunlight regimes and extremely warm temperatures showed a fast vegetative growth and even higher survival, but their reproductive capacity was completely nullified (Fig. 1; Table 2). Such a trend indicates that in the short term, the different sunlight regimes, and extremely warm temperature thresholds, promote a deep alteration in plants phenological responses. Likely, this contrasting response could be strongly linked to different ecophysiological ability within plants to quickly adapt to the extreme growing conditions above ground.

### Photosynthetic acclimation traits

Photosynthesis of many C_3_ plants responds linearly to low light intensities, but rapidly towards the 500 μmol m^−2^ s^−1^ of irradiance (i.e. 25 % of total possible irradiance PPFD = 2000 μmol m^−2^ s^−1^), the linear relationship between absorbed quanta and photosynthetic assimilation begins to plateau, to then decline if the irradiation continues to increase to saturation levels (Marino *et al.* 2010). In this study, interesting photosynthetic acclimation traits are highlighted. The photosynthetic curves in response to light suggest that the maximum CO_2_ assimilation (*A*_max_) saturates rapidly at low irradiance flux between 12 and 24 % (PPFD_50_), remain saturated towards an intermediate irradiance flux between 25 and 50 % (PPFD_95_), until its decline to a high irradiance flux >60 %. Wild versus cultivated plants have higher photosynthetic capacity traits, because their CO_2_ assimilation capacity and electrons transport efficiency were higher towards the asymptotic trajectory of photosynthetic curves ~500 μmol m^−2^ s^−1^ (Fig. 2; Table 3). Due to that *A*_max_ in response to high irradiance flux can be limited both by Rubisco activity and TPU, the calculated values for maximum electrons transport (*J*_max_) could not reflect their maximum possible rate. In this study, following the recommendations provided by Buckley and Diaz-Espejo (2015), to avoid ambiguity for *J*_max_ (Fig. 2), we presented the value of irradiance at which *J*_max_ was reached. Namely, *J*_1000_ and *J*_1400_ for leaves from wild and cultivated plants, respectively (Table 3).

In addition to *A*_max_ and *J*_max_, other photosynthetic traits are highlighted. Data set suggests that wild plants could maximize their CO_2_ assimilation even at low light levels, since the dark respiration (*R*_dark_) was overtaken at a low irradiance flux; therefore, wild versus cultivated plants could have a lower LCP (Fig. 2; Table 3). The low LCP, higher photosynthetic capacity and electrons transport efficiency recorded in wild plants could be related to an efficient acclimation strategy for maximize the sunlight photosynthetic capture at shade gradients in the understory. By contrast, data set suggests that under extreme cultivation conditions, plants could have lower photosynthetic capacity per each quantum absorbed. Besides, the higher LCP recorded in cultivated plants suggests that respiration could be a significant limiting factor for carbon gain when light availability is lower. Regardless the different LCP, the optimal functioning of photosynthesis could occur in a low to intermediate range of irradiance.

The negative growth response and almost null survival recorded in plants grown in high sunlight regimes and extremely warm temperatures (Fig. 1; Table 2) could be attributable to an abrupt disruption of photosynthetic acclimation mechanisms caused by the excessive photonic energy and extreme thermal thresholds. This specific hypothesis is partially supported by the rapid photosynthetic saturation at low irradiance and photosynthetic declining trend to high irradiance (Fig. 2; Table 3), as well as by different studies about photosynthetic acclimation (Zhu *et al.* 2018; Fernández-Marín *et al.* 2020). The functional traits recorded in cultivated plants under different sunlight regimes and extremely warm temperatures clearly suggest that their acclimation plasticity depends both on biophysical regulation and photosynthetic capacity traits (Table 4). They efficiently cope heat stress through higher stomatal conductance that at expense of excessive water loss, minimize the risk of heat damage on their photosynthesis through cooling by high transpiration and inner cooling mechanisms, because leaf temperature reached very warm thresholds even under the shade (Table 4). This hypothesis is well supported by different studies addressing the causes and consequences of photosynthetic thermal acclimation (Liguori *et al.* 2017; Niinemets 2018).

A recent study shows that maintenance of non-lethal temperature in leaves imposes constraints on stomatal regulation (Blonder and Michaletz 2018). Our data suggest that the contrasting CO_2_ diffusion traits (Table 4) could represent not only the reflection of different gas exchange traits by themselves, but also an efficient photosynthetic response to the exposure of leaves to extremely warm temperatures (Niinemets 2018; Zhu *et al.* 2018). Studies suggest that plant species whose photosynthesis responds at low intercellular CO_2_ concentration tend to display a high ratio between the mesophyll and stomatal conductance. Under such conditions, an improvement in the photosynthetic water use and positive carbon assimilation could prevail, given that stomatal limitation may be exacerbated especially in phenotypes adapted to shade (Flexas *et al.* 2013; Martins *et al.* 2014). In this study, the averages from stomatal and mesophyll conductance recorded (Table 4) are like the ranges recorded in other C_3_ species (Flexas *et al.* 2008; Martins *et al.* 2014).

The multiple correlation analysis suggests that the increase of maximum CO_2_ assimilation at elevated CO_2_ concentrations and warm leaves temperature in plants grown under intermediate sunlight regimes (Table 4) could strongly depend on the simultaneous interplay of several traits: for instance, the increase of electrons transport and nitrogen allocation, and others (**Supporting Information B**). The different photosynthetic capacity and the lower photosynthetic water use efficiency recorded in cultivated plants (Table 4) could be traits related to the functional acclimation for maximizing CO_2_ assimilation and carbon gain in response to the harsh growing conditions. Similarly to the findings of Martins *et al.* (2014), our data suggest that at different irradiance flux and warm temperatures, the net CO_2_ assimilation could be constrained mostly by the Rubisco activity, since the estimated chloroplastic CO_2_ concentration was lower than those in the transitory point towards the Rubp regeneration in curves, which is mostly regulated by the electrons transport rate and trioses utilization rate (Fig. 3; Table 4). Intriguingly, although the low sunlight constrained the photosynthetic capacity of plants, the ratio between the electrons transport rate and Rubisco carboxylation velocity was similar in wild and cultivated plants under different sunlight regimes (Table 4).

Data set suggests that the carbon economic spectrum of leaves could be significantly altered in response to intermediate sunlight regimes and extremely warm temperatures, because plants increase their respiratory level and consequently the ratio between respiration and assimilation (Table 4). Several closely related metabolic process during photosynthesis and respiration could simultaneously improve plants performance specifically when they grow under intermediate sunlight regimes and extremely warm temperatures. This hypothesis is partially supported by our multiple correlation analysis (**Supporting Information B**). Carbon and nitrogen economic spectrum recorded in plants under extreme cultivation conditions could be explained not only by the significant interplay between respiration and specific photosynthetic capacity traits (Tables 4 and 5), but also complementary metabolic processes, e.g. production of carbon precursors, redox balancing, among others (O’Leary *et al.* 2018; Scafaro *et al.* 2021).

The displacement of CO_2_ compensation and photoco­mpensation points recorded in photosynthetic curves from cultivated plants (Fig. 3; Table 4) could be strongly influenced not only by environmental conditions and CO_2_ diffusive constraints, but also by the interplay between respiration rate, photosynthetic capacity and photosynthetic nitrogen economy (Tables 4 and 5). This hypothesis is strongly supported by studies which show that carbon gain is enhanced by alternate metabolic pathways that feed the CO_2_ reassimilation by Rubisco enzyme through recycling mechanisms of photorespired and respired CO_2_ (Busch 2020; Busch *et al.* 2020). It is expected that under a warmer climate with rising CO_2_ levels (https://climate.nasa.gov/vital-signs/carbon-dioxide), natural selection may differentially favour those plants genotypes and phenotypes which have thermal and photonic tolerance in their cell walls, membranes and their proteins for provide stability and efficient balance between the CO_2_ diffusion, assimilation, respiration, photorespiration and recycling rate of photorespired and respired CO_2_ (Niinemets 2018; Clemente-Moreno *et al.* 2019).

### Architectural, anatomical and biochemical traits

Differential expansion of leaf surface enables ecophysiological acclimation to shifts on sunlight regimes or shade gradients (Madeline *et al.* 2012). To maximize their sunlight photosynthetic interception surface, plants grown at low sunlight regimes could simultaneously increase the specific, individual and total leaf area with respect of total biomass (Table 5), but at expense of developing thinner leaves with a low mass per area (Table 5). Besides, the multiple correlation analysis suggests that the increase of photosynthetic interception surface negatively correlates to all photosynthetic capacity traits (**Supporting Information B**). Studies show that as the sunlight regimes increase, plants develop thicker leaves with extra palisade cells layers, containing thousands of chloroplasts and photosynthetic enzymes, which consequently enhance the photosynthetic capacity per unit of leaf area (Poorter *et al.* 2019). Under intermediate sunlight regimes, the balance between architectural and leaf structural traits could be linked to a cost-effective functional strategy because plants modify their photosynthetic assimilation surface, while prioritize mass gain in leaf, since photosynthetic capacity significantly increases (Tables 4–6).

The multiple correlation analyses suggest that the increment of leaf mass per area is strongly linked to increasing of abaxial stomatal index and density (Table 5 and **Supporting Information B**). It has been shown in other studies, the simultaneous increase of leaf mass per area and stomatal density could be also strongly linked to modifications of other anatomical, metabolic and biophysical parameters (Martins *et al.* 2014; Clemente-Moreno *et al.* 2019). It could be also related to a systemic stimulus promoted by changes in the spectral composition of sunlight and thermal acclimation (e.g. Red/Far-red ratio plus warm temperatures and its effect in phytochromes signalling for morphogenesis) (Jung *et al.* 2016; Matthews *et al.* 2020). Data set shows that plants grown under intermediate sunlight regimes increase the photosynthetic pigment content. Pigments are functional ecophysiological indicators of plants acclimation light availability (Lichtenthaler and Buschmann 2001; Liguori *et al.* 2017).

Plants grown under intermediate sunlight regimes allocated a lower ratio of *chlorophyll a* and *chlorophyll b* (~1.5), while those under low sunlight regimes (~2.1) (Table 5). The higher content of chlorophylls, xanthophylls and carotenoids in plants grown under intermediate sunlight regimes and extremely warm temperatures (Table 5; **see****Supporting Information A—Table S2**) suggests a systemic effect on the light-harvesting protein complexes, the reaction centres and the inner energy dissipation mechanisms of excessive thermal and photonic energy (Lichtenthaler and Buschmann 2001; Liguori *et al.* 2017). The increase of photosynthetic pigments under the intermediate sunlight regimes may occur along with other intriguing biochemical modifications such as the nitrogen partition towards photosynthetic components (Table 5). The total fraction of nitrogen per unit of leaf area and the proportional fraction allocated to carboxylation, bioenergetics and light-harvesting are like findings of other studies (Niinemets and Tenhunen 1997; Niinemets 2007).

Consequently, the proportional nitrogen partition recorded (Table 5) could reflect the actual nitrogen economy in leaves and thus, lends support to explain why photosynthetic performance of plants grown under intermediate versus low sunlight regimes could be remarkably different (Table 4). However, from the total nitrogen allocated to photosynthetic protein complexes, a higher amount of nitrogen allocated to both carboxylation and bioenergetics could play a central role for carbon gain since they significantly correlated to Rubisco carboxylation velocity, electrons transport rate, TPU rate and respiration (see **Supporting Information B**). Photosynthetic performance also depends on alternate metabolic pathways (enzymatic or non-enzymatic), which involve the translocation and storage of carbon, hydrogen, nitrogen and multiple mineral nutrients (Niinemets 2007; Szabò and Spetea 2017). The multiple correlation analysis (see **Supporting Information B**) suggests that increase of several and specific nutrients (Table 5) could be mostly explained by the nitrogen allocation to light-harvesting protein complexes. Besides the significant correlations between the increase of iron in leaves and the increase of phosphorous and copper (Table 5) could related to the accumulation of ferritin in the chloroplasts, because this biochemical process directly responds to light availability, circadian clock and signalling pathways which regulate the nutritional homeostasis of iron and phosphorous (Bournier *et al.* 2013).

### Growth rate and biomass allometry

In general terms, plants grown at low sunlight can fix a relatively fewer amount of carbon during photosynthesis due to lower stomatal conductance and lower photosynthetic water use efficiency; consequently, biomass gain and nutrient requirement are lower (Poorter *et al.* 2012c, 2015). Therefore, according to the functional equilibrium model, plants grown at a low light level should show higher biomass allocation to stems and especially leaves at the expense of roots (Poorter *et al.* 2012c, 2015). Our data set is consistent with such principle because the lower photosynthetic capacity of plants grown in low sunlight regimes (Table 4) was reflected in their low biomass gain and their significant allometry towards stems and leaves at the cost of less investment in roots development (Tables 5 and 6). Therefore, the growth strategy of plants under low sunlight regimes is to lengthen their stems maximizing leaves expansion (Tables 5 and 6), compensating by this way the gas exchange restrictions and lower photosynthetic capacity (Tables 4 and 5).

By contrast, the multiple correlation analysis suggests that under intermediate sunlight regimes, the increase of growth rate, biomass gain and reproductive mass fraction significantly depend on the rise of most important photosynthetic capacity traits, respiration rate and the enhancement of the nitrogen economy in leaves (see **Supporting Information B**). The impulse of the above-ground and below-ground vegetative biomass gain, as well as the reproductive development recorded in plants grown under intermediate sunlight regimes and extremely warm temperatures could also be attributed to a systemic response to the spectral composition of sunlight regimes (i.e. Red/Far-red ratio) and thermal thresholds, since recent studies suggest that the spectral quality of light and temperatures activate the phytochromes triggering signalling pathways for promoting the above-ground and below-ground development (vegetative and reproductive growth plus roots development) (Jung *et al.* 2016).

### Principal components analysis

Determination of most sensitive indicators during plants development is fundamental to evaluate the conditions of plants in ecological systems. The principal components analysis is an effective statistical tool to reveal meaningful ecophysiological or biochemical parameters linked to acclimation plasticity and adaptive strategies among plants species when thriving in different environmental contexts (Füzy *et al.* 2019). The principal components analysis suggest that the main explanatory traits in response to the low sunlight regimes could be the specific leaf area, and some traits related to CO_2_ diffusion, e.g. the stomatal limitation, the mesophyll conductance and the chloroplastic CO_2_ concentration (Fig. 4). By contrast, the main explanatory traits in response to intermediate sunlight regimes could be the photosynthetic pigments, the nitrogen allocation to light-harvesting and carboxylation, the Rubisco *V*_cmax_, stomatal conductance, total biomass gain, among others. The significant traits recorded (Tables 4–6), as well as the main explanatory traits extracted by the principal components analysis (Fig. 4) could be useful in comparative studies about acclimation strategies among different C_3_ species adapted to similar or different habitats.

## Conclusions

Under the extreme heat of Sonoran Desert, by using shading nets for plants cultivation, and where water and nutrients availability was not a stress factor, dataset suggests that low, intermediate and high sunlight regimes could drive significant alterations on the survival rate, growth, reproductive capability and functional acclimation traits. This study provides multiple reference traits from a C_3_ species and useful insights for future research about functional plasticity traits to cope with environmental stress factors occurring in semi-arid habitats under the context of a warmer climate and rising CO_2_ levels.

## Supporting Information

The following additional information is available in the online version of this article—

**Supporting Information A.**

**Figure S1.** Cultivated plants.

**Figure S2.** Summary of weather conditions at open sky during cultivation of plants.

**Figure S3.** Stomatal density.

**Table S1.** Physical and chemical profile of the experimental soil.

**Table S2.** Sunlight regimes and air temperatures thresholds.

**Table S3.** Summary of soil temperatures.

**Table S4.** Results of the analysis of variance of the photosynthetic traits.

**Table S5.** Photosynthetic parameters derived from the *A*/*C*_c_ curves fitting.

**Table S6.** Results of analysis of variance of photosynthetic traits.

**Table S7.** Results of the analysis of variance of architectural, anatomical and biochemical traits.

**Table S8.** Results of the analysis of variance of growth traits.

**Supporting Information B.** Results of the principal components analysis and multiple correlation analysis; as well as the raw data set of photosynthetic curves.

plac017_suppl_Supplementary_Material

plac017_suppl_Supplementary_Data

## Data Availability

The raw data are available in **Supporting Information B** (excel file). Please contact the author for additional data requests, and the data will be made available upon your request.

## Notes

**Form & Function. Chief Editor**: Kate McCulloh
