# Supplementary material for: Functional plasticity of Capsicum annuum var. glabriusculum through multiple traits
Source: AoB Plants. 2022 May 5;14(3):plac017. doi: 10.1093/aobpla/plac017 (PMC9237842; doi:10.1093/aobpla/plac017)
Supplement: plac017_suppl_Supplementary_Material [file plac017_suppl_supplementary_material.docx]

**Supporting Information A** from the article:

**Functional plasticity of *Capsicum annuum* var. *glabriusculum* through multiple traits**

**Materials and Methods (Full description)**

**Climatological traits of the experimental site**

In semiarid environments such as the Sonoran Desert, the bimodal patterns of precipitations cause fast climatic changes during and between the growing seasons (CONAGUA 2014). The seasonal trends of climate are characterized by extreme conditions depending on the latitudinal, longitudinal, and altitudinal gradients and variations of cloudiness. During late spring, summer, and early autumn prevail the high sunlight regimes (35-64 mol mˉ²dˉ¹), air temperatures thresholds from warm to very warm (25-48°C), moderate to high relative humidity and rainfalls (22-60%, 74-157 mm); whereas, during late autumn, the winter, and the early spring prevail the low sunlight regimes (21-36 mol mˉ²dˉ¹), warm to cold air temperatures thresholds (30 to -6°C), moderate to high relative humidity and light rainfalls (22-48%, 24-40 mm) (CONAGUA 2014). Specifically for this experiment, we recorded the daily sunlight regimes and their air temperature thresholds during part of the spring and throughout the summer (Table S2). In addition, as a climatological reference, we provided the data from the air relative humidity and rainfalls throughout the experiment (Fig. S2).


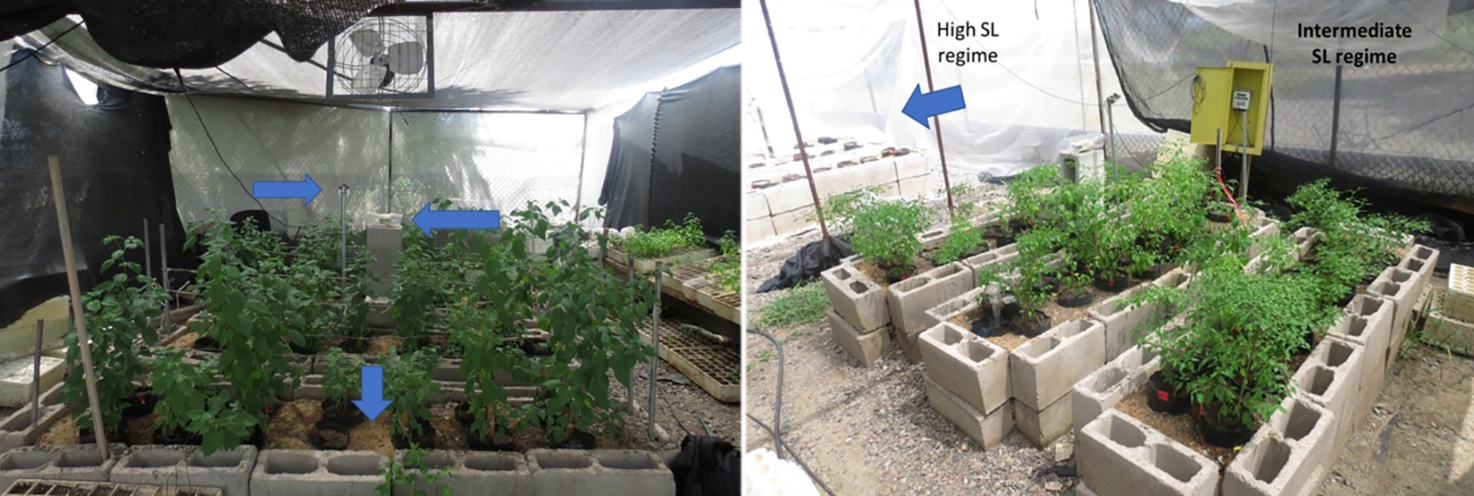


**Figure S1.** Plants grown in low sunlight regimes (Left image) and intermediate sunlight regimes (Right image). Blue arrows show the sensors (left) and the high sunlight regime (right).

**Modification of sunlight regimes to differentially shade the plants**

For this experiment, we used crystalline-colored anti-aphids mesh, and different polyethylene black meshes to differentially shade the plants. To evaluate the effect of the whole range of the photosynthetically active photons flux density (i.e. PPFD= sunlight regimes in µmol mˉ²sˉ¹ or in mol mˉ²dˉ¹), we modified levels of exposure to provide low, intermediate, and very-high sunlight regimes. We cultivated plants under the shading nets set to provide low, intermediate, and very-high sunlight regimes (henceforth referred to as treatments). The daily photosynthetically active photons flux density (i.e. PPFD= sunlight regimes in mol mˉ²dˉ¹) was recorded in each treatment by using a WatchDog™ Mini-station. Namely, throughout plants development during part of spring, and throughout summer, the sunlight regimes (mol mˉ²dˉ¹) were daily recorded at the open sky and under the different shading nets (treatments). To record the warming effect caused by the shading nets, the daily air temperature thresholds (minimum and maximum temperatures, °C) were recorded, both at the open sky and under the shading nets (Table S2).

To modify the sunlight exposure levels, we built a rectangular frame (with steel rectangular tubes: 5 m width × 2 m high × 17 m length) covered by shading nets. We used crystalline polyethylene anti-aphids nets and two different polyethylene black nets to differentially shade plants. The low sunlight was established using crystalline polyethylene anti-aphids nets (i.e. CPAN 40 × 25 threads per square inch) plus a black color high-density polyethylene nets (BPN 44 × 14 threads per square inch), both placed on the ceiling and walls. Adjacent, the intermediate sunlight was established using the CPAN 40 × 25 plus BPN 25 × 25, both placed on the ceiling and walls. Right next, the high sunlight was established using only the CPAN 40 × 25 placed on the ceiling and walls. The CPAN virtually does not provide shade because its crystalline-colored. On the contrary, according to the mesh manufacturers and local distributors ([www.hydroenvironment.com](http://www.hydroenvironment.com)), the BPN 25 × 25 and the BPN 44 × 14, provides ⁓50-95% of shade respectively.

To know with accuracy the seasonal variations of sunlight both inside and outside (i.e. open sky) the shading nets, the instantaneous/daily photosynthetically active photon flux density was recorded (i.e. PPFD= µmol mˉ²sˉ¹, mol mˉ² dayˉ¹ respectively; WatchDog Spectrum Technologies™). Three round sensors (Light-Scout Quantum Light Sensor™) were placed at the height of the plants' canopy (85 cm) and right in the middle of each level of sunlight exposure towards the plants. An additional sensor was placed outside the shading nets (i.e. in the open sky without any shade). The sunlight regimes (µmol mˉ²sˉ¹→ mol mˉ²dˉ¹) were recorded daily throughout plants development during part of spring and throughout summer (157 days in total, Table S2). Plants were cultivated in the low, intermediate, and high sunlight regimes.

The crystalline polyethylene anti-aphids nets did not provide shade, but the different polyethylene black nets allowed different sunlight penetration towards plants. The shading nets set together have three main advantages: 1. They avoid insect pests during cultivation (e.g. aphids or white-fly which are vectors of viral diseases in *Capsicum* spp.); 2. They allow a differential irradiance penetration towards plants, and 3. They allow water coming from the rainfalls to drop freely towards plants. One disadvantage of using different shading nets for plants cultivation is that the airflow speed which passes through them can decrease; this, in turn, can promote the significant increase of air temperature experienced by plants (Table S2). We used a small electric ventilation fan during this experiment just to promote air circulation around plants, but not to diminish air temperatures. Therefore, the data from air temperatures reflected the extreme natural thresholds experienced by plants in such conditions under the shading nets. Under the shading nets plants were exposed to the natural climatic conditions throughout their development (i.e. natural sunlight, temperatures, air relative humidity, and precipitation).

**Seeds source, germination protocol, and plants acclimation before transplanting**

The experiment started in mid-spring (April 18-2016) and lasted 157 days towards the end of summer. Seeds obtained from harvested fruits in a previous experiment were used and sowed. Seeds were hydrated in distilled water for 24 h and afterward, they were treated for 24 h with gibberellic acid (500 ppm of Biogib™). Afterward, seeds were sown (April 18-2016) in a wet mixture of sterilized river sand/peat moss (1:1 v/v; 26% of gravimetric moisture) placed in nine polystyrene trays (60 cavities each) until transplanting to pots. The nascent plants were kept in the germination trays to acclimatize them until reaching a suitable size for transplantation.

For suitable acclimatization to sunlight levels, three germination trays with the nascent plants were kept in the low sunlight, and such plants were used exclusively for transplanting and cultivating them right there. The other six germination trays with the nascent plants were kept under intermediate sunlight, and such plants were used exclusively for transplanting and cultivating them right there under intermediate or high sunlight. During the germination and acclimation stage, we avoided placing germination trays in high sunlight regimes. This was done to protect plants from excessive irradiance, high evapotranspiration, and heat stress in the early growth stage. Therefore, we allow them to reach a suitable leaf area before the transplant (153±48 cmˉ²mˉ² equivalent to 49±7 leaves). Plants were watered daily to maintain constant moisture in the soil mix (26% of gravimetric moisture).

Additionally, to obtain good size plants in the short period of germination and acclimation (1.5 months), the emerging plants were watered every seven days with a 0.1% solution of macro/micro-nutrients (Miracle Gro™ 15% N, 30% P₂O₅, 15% K₂O, 0.02% B, 0.07% Cu, 0.15% Fe, 0.05% Mn, 0.0005% Mo, 0.06% Zn). Fertilizer concentration and frequency of application were based on the recommendation of the manufacturer Miracle Gro™. Except on rainy or cloudy days, plants were watered daily (only tap water without chemical nutrients) to maintain constant moisture in the soil mix (26% of gravimetric moisture). With this germination protocol and with the seeds batch used, we obtained a germination rate of 33±10%. 49 days after sowing, plants of homogeneous size were transplanted to the different sunlight levels.

**Table S1.** Physical and chemical profile of the experimental soil. Reference values correspond to sampled soil from wild *Capsicum annuum* var. *glabriusculum* plants. *Averages and standard deviation from four composite samples.

| **Physical profile of the experimental soil** | | | |
| --- | --- | --- | --- |
| Taxonomy | Averages  values | Reference  values | Units |
| sandy clay | 5 | 7 | % |
| Silt | 9.5 | 18 | % |
| Sand | 85.5 | 75 | % |
| Organic matter | 4 ± 2 | 3.6 ± 2.3 | % |
| **Fertility profile of the experimental soil** | | | |
| Traits | *Average value | Reference value | Units |
| pH | 6.6 ± 0.3 | 6.9 | - |
| Electric conductivity | 2.6 ± 0.2 | 1.3 | dS m^-1^ |
| N-NO₃¯ | 90.7 ± 64.9 | 25.4 | mg kg^-^¹ |
| P-PO₄¯ | 81.5 ± 57.5 | 13.6 | mg kg^-^¹ |
| K | 164.3 ± 65.7 | 110 | cmol kg^-^¹ |
| Ca | 4000 ± 1293 | 2970 | cmol kg^-^¹ |
| Mg | 430 ± 69.7 | 210 | cmol kg^-^¹ |
| S | 41 ± 21 | 25 | mg kg^-^¹ |
| Fe | 22.5 ± 22.4 | 20.5 | mg kg^-^¹ |
| Cu | 1.4 ± 0.9 | 1.1 | mg kg^-^¹ |
| Zn | 1.3 ± 0.9 | 10.6 | mg kg^-^¹ |
| Mn | 25.2 ± 15.5 | 46.7 | mg kg^-^¹ |
| Na | 23 ± 5.4 | 56.4 | mg kg^-^¹ |

**Soil traits and transplanting to treatments**

The soil bulk mixture used in this experiment was river sand and native soil collected from wild plants. The river sand and the soil collected from wild plants contained different native arbuscular mycorrhizal fungi (and additional microbiota), and were used previously to cultivate plants without any chemical fertilization (data not shown). Therefore, plants were cultivated in native soil with biological, physical, and chemical characteristics analogous to those in which wild plants develop (Table S1). The soil mixture was placed in perforated plastic pots (n= 108 pots of 24 × 35.5 cm and 9 kg capacity, volume= ~16,059 cm³). The soil mixture was moistened to saturation. The soil volume was enough to allow adequate plants development to study their biomass gain/allometry (i.e. enough roots growth, and adequate stems, leaves, and fruits development). The pots were placed right in the middle of each sunlight exposure level at a distance of ~30cm from each other. This allowed that all leaves of each plant received a similar incidence of sunlight during each photoperiod; thus avoiding the shade of one plant towards another. 49 days after sowing, plants of homogeneous size were transplanted to pots placed at each sunlight exposure level: n= 36 plants in the low sunlight, n=36 plants under the intermediate sunlight, and n=36 plants in the high sunlight.

**Growth conditions**

The experiment lasted 157 days in total. Seeds sowing, germination stage, and plants acclimation stage occurred from mid-spring until the end of spring (i.e. From April 18, May, until June 6). The transplantation and development stage occurred at the end of spring, and full development during summer (i.e. June, Jul, Aug, Sept). All plants in their pots were arranged parallel to the solar path (Fig. S1). Additionally, to avoid stress by excessive heat diffusion towards the rhizosphere, all pots were buried at a depth of ~30 cm using gravel and ~5cm of wood sawdust. Throughout the experiment, we measured the soil temperatures (manual Taylor™ thermometer) at a depth of 7.5 cm in two pots per each sunlight exposure level. Soil temperatures were measured between 2:00 and 3:00 pm to obtain the highest temperature records during each photoperiod, since approximately at such hours the highest peaks of air temperatures were recorded (data not shown). As mentioned before, to evaluate the light availability at each exposure level (i.e. the low, intermediate, and high sunlight regimes; and at the open sky), the daily variations of the photosynthetically active photons flux density (PPFD= µmol mˉ²sˉ¹, mol mˉ² dayˉ¹) were recorded (WatchDog Spectrum Technologies™; Table S2). Besides, the daily variations of air temperature at the open sky were recorded at 15 min intervals throughout plants development.

The HOBO™ microstations were placed near plants by using a polystyrene base to protect them from excessive heat. Additionally, the HOBO microstations were covered with a plastic bottle with hollows on the sides to protect them from rains and at the same time allow free airflow. Reference data of seasonal variation of rainfalls/relative humidity were taken from nearest climatological stations (<http://www.siafeson.com/remas2/index.php/tablero>; CIAD microclimate station). The summary of seasonal climatological conditions at open sky throughout this experiment is shown in Fig. S2. Photographs of plants are shown in Fig. S1.

After transplantation, plants fully grew throughout the summer under the influence of natural variations of climatic conditions. Since plants have thin leaves they are very sensitive to water stress in soil. Namely, in a matter of minutes, water stress in soil plus transpiration causes a progressive loss of leaves turgidity and if extended to a critical point, leaves lose functionality in an irreversibly way, and eventually, they detach from stems (data not shown). Therefore, to completely avoid water stress throughout plants development, after transplanting and except on cloudy or cloudy/rainy days, we applied daily irrigation (500 mL per plant) mainly in the warmest and sunny days during summer. This was done to compensate for the high evapotranspiration and thus maintain constant levels of moisture in the soil (i.e. ≤3 kPa of moisture pressure in soil measured by Irrometer™). Besides, after transplanting, we did not apply chemical fertilization since the native soil used provided adequate levels of macroelements and microelements for plant development at least for one growing cycle (Jiménez-Leyva *et al*. 2017; Table S1). This means that throughout plants' development there was no water or nutrients stress. After plant transplanting, some weeds and/or other plants that germinated in the experimental soil were immediately eliminated to avoid competition for soil resources and thus obtain the maximum possible growth during the experiment.

**Record of air temperature thresholds and heat sum throughout plants phenological development**

The daily minimum and maximum air temperature thresholds (°C) were recorded in the different sunlight regimes and open sky (HOBO™ microclimatic stations and CIAD station). The heat sum was quantified throughout the plants phenological development during summer. The degrees days were calculated using a base temperature (10°C) and the records of daily maximum and minimum air temperature thresholds, according to the equations proposed by Moreno *et al*. (2014). Plants phenological transition in the seasonal timeline was recorded (Table 2).

**Measurement of plants survival rate**

Three days before gas exchange measurements, survival rates (%) of plants were quantified at the stage of full vegetative and reproductive growth. This was conducted by counting the alive and dead plants in the treatments (n= 36 per treatment). The main purpose was to estimate the short term impact of sunlight regimes on plants survival; in such a way that it would let us know the number of plants available to perform enough repetitions for the statistical validation of all the measurements described. Taking into account only the plants survivors of each treatment and before making the measurements, a random draw was made for the choice of the individuals to be evaluated.

**Gas exchange measurements**

Even though there was not water or nutrients stress, in the high sunlight very few plants survived (Table 2). Therefore, all measurements were conducted only in plants grown in low and intermediate sunlight. The gas exchange measurements refer to CO_2_ assimilation curves in response to the photosynthetic photons flux density (A/PPFD) and CO₂ concentrations (A/Ci). The main purpose of gas exchange measurements was to take a snapshot of plants' photosynthetic traits just when they go through the full vegetative and reproductive growth. Therefore, all gas exchange measurements were conducted at this phenological stage. To get reference values, previous to this study, we conducted gas exchange measurements in leaves from wild plants. Afterward, in this experiment, we conducted the gas exchange measurements in plants grown in low and intermediate sunlight. Plants were grown in native soil with good availability of mineral nutrients and they were constantly watered throughout the experiment, maintaining a moisture partial pressure in the soil ≤3 kPa (Irrometer™). Therefore, plants did not suffer from water or nutrients stress at any time, much less when the gas exchange measurements were carried out. Through this strategy, we ensure the non-interference of other variables; therefore, plants exhibited their maximum photosynthetic potential under extreme experimental conditions. All the standard symbols and abbreviations (their units and definitions) corresponding to gas exchange traits that are mentioned in the text are shown in Table 1.

All the A-PPFD and A/Ci curves were measured on fully-expanded mature (˃6cm²), healthy, and completely turgid leaves from a branch in the upper canopy layer. From wild plants, we measured four A-PPFD and four A/Ci curves (in a single day). From the cultivated plants, we measured six A-PPFD curves and six A/Ci curves per treatment. All gas exchange measurements from cultivated plants were completed in two consecutive days under completely sunny conditions i.e. one day for all the A-PPFD curves and another for all the A/Ci curves. The A-PPFD and the A/Ci curves were measured before midday and onwards because during those hours the leaves are exposed to maximum sunlight/temperatures (data not shown). The raw gas exchange dataset is shown in supporting Information B.

Measurements of gas exchange in wild plants were conducted towards end of summer and the beginning of autumn just at the period when plants go approaching to their maximum development. Four wild plants were selected in their natural habitat (“La Cieneguita”, Baviácora, Sonora México, altitude: 980 masl, 29.32361 LN, -110.00922 LW; Fig. 13). The gas exchange measurements (A-PPFD and A/Ci curves) were conducted using a single portable infrared gas analyzer system following standard procedures (LICOR 6400XT™, manual-LICOR™; Rodeghiero *et al*. 2007). Before the gas exchange measurements, each point indicated in the preparation checklist was reviewed according to standard protocols (LICOR 6400XT™). The stomatal ratio was set as 1 because the stomata of leaves are mainly distributed on the abaxial surface, so that the effect of some stomata which occur in the adaxial surface is negligible. Measurements were conducted once the steady-state of CO₂ and water vapor exchange were achieved in the IRGA (i.e. Coefficient of variation ˂1%, LICOR 6400XT™). The CO₂ sensors and water vapor sensors of the system were calibrated using the gas device (i.e. 21% oxygen and nitrogen as balance) and the humidification system, respectively (LI-610 Portable Dew Point Generator, LICOR™).

During gas exchange measurements, leaves temperature followed its natural course as a function of the ambient temperature, but the irradiance (i.e. PPFD= µmol mˉ²sˉ¹) levels were provided by the red-blue light source device (50:50 6400-02B™ LED light source). Due to the extremely warm conditions plants experienced (Table S2), we had some difficulties (i.e. more waiting time to measurements of assimilation rate at each *CO*₂ level) to match the IRGA and stabilize the parameters inside the chamber (i.e. Coefficient of variation ˂1%). To solve this, we fixed the flow rate in 500 μmol mˉ²sˉ¹ to eliminate the diffusion effect. The diffusion and leakage check (i.e. the leakage of *CO*₂ and water vapor diffusion into and out chamber) were conducted as described by Rodeghiero *et al*. (2007) and LICOR 6400-XT manual (LICOR™).

The *A*-PPFD curves from wild plants were measured under the following conditions: 1. Flow: 500 μmol mˉ²sˉ¹, 2. *CO*₂ concentration in the chamber 400 μmol molˉ¹, 3. Leaves temperature 34±0.5⁰C, 4. Relative humidity inside chamber 25±2%, and 5. VPD= 4±0.2 kPa. The PPFD (μmol mˉ²sˉ¹) levels were programmed in the following order: 2000, 1500, 1000, 500, 400, 300, 200, 100, 50, 20 and 0. The *A*-PPFD curves from plants grown under intermediate sunlight were measured under the following conditions: 1. Flow: 500 μmol mˉ²sˉ¹, 2. *CO*₂ concentration 400 μmol molˉ¹, 3. Leaves temperature 39±2°C, 4. Relative humidity inside chamber 65±7 %, and 5. VPD= 2.0±0.7 kPa. The PPFD (μmol mˉ²sˉ¹) levels were programmed in the following order: 2000, 1500, 1000, 500, 400, 300, 200, 100, 50, 20 and 0. The *A*-PPFD curves from plants grown in low sunlight were measured under the following conditions: 1. Flow: 500 μmol mˉ²sˉ¹, 2. *CO*₂ concentration 400 μmol molˉ¹, 3. Leaves temperature 36±2°C, 4. Relative humidity inside chamber 63±0.7%, and 5. VPD= 2.2±0.1 kPa. The PPFD (μmol mˉ²sˉ¹) levels were programmed in the following order: 2000, 1500, 1000, 500, 400, 300, 200, 100, 50, 20, and 0. In each *A*-PPFD curve, the average waiting time for *CO*_2_ assimilation measurement at each PPFD level was: ̴120 seconds per light level.

Wild plants naturally grow in a heterogeneous sunlight environment caused by the multiple gaps in the canopy of trees that provide a partial shade. Before the *A*/*C*_i_ curves measurements, we measured the natural light environment of wild plants. This was done so that the *A*/*C*_i_ curves were programmed to the average light level leaves naturally intercept. With a linear quantum sensor (LI-191SA, LICOR™), we measured the instantaneous sunlight irradiance in wild plants (i.e. PPFD= μmol mˉ²sˉ¹). At the height of the upper canopy (1 m), we placed the LI-191SA sensor in different positions (i.e. until covering an angle of 180° per plant), to measure the incident sunlight levels. An average of PPFD= 404±347 μmol mˉ²sˉ¹ was recorded (n= 4). Therefore, all *A*/*C*_i_ curves measured in wild plants were programmed at PPFD= 400 μmol mˉ²sˉ¹ as described further. Each *A*/*C*_i_ curve consisted of at least 13 different *C*_a_ values. Each curve took approximately 45 min to complete. The *A*/*C*_i_ curves from wild plants were measured under the following conditions: 1. Flow= 500 μmol mˉ²sˉ¹, 2. PPFD= 400 μmol mˉ²sˉ¹, 3. Tleaves= 32.7±0.9 ⁰C, 4. Relative humidity inside chamber= 40±2 %, and 5. VPD= 2.8±0.09 kPa. The *CO*₂ levels were programmed in the following order: 400, 300, 200, 100, 50, 400 (as a checkpoint), 600, 800, 1000, 1200, 1400, and 1600 μmol CO₂ mˉ²sˉ¹.

The *A*/*C*_i_ curves from cultivated plants were measured using the light levels (PPFD= μmol mˉ²sˉ¹) near to the maximum instantaneous irradiance recorded in each exposure level (Table S2). The *A*/*C*_i_ curves from plants grown in low sunlight were measured under the following conditions: 1. Flow = 500 μmol mˉ²sˉ¹, 2. PPFD= 94±0.4 μmol mˉ²sˉ¹, 3. Tleaves= 40± 2⁰C, 4. Relative humidity inside chamber= 56±0.6%, and 5. VPD= 3.09±0.8 kPa. The *CO*₂ levels were programmed in the following order: 400, 300, 200,175, 150, 125, 100, 400 (as a checkpoint), 500, 600, 800, 1000, and 1200 μmol CO₂ mˉ²sˉ¹. The *A*/*C*_i_ curves from plants grown under intermediate sunlight were measured under the following conditions: 1. Flow= 500 μmol mˉ²sˉ¹, 2. PPFD= 648±4 μmol mˉ²sˉ¹, 3. Tleaves= 40± 2⁰C, 4. Relative humidity inside chamber= 70±6%, and 5. VPD= 1.9±0.75 kPa. The *CO*₂ levels were programmed in the following order: 400, 300, 200,175, 150, 125, 100, 400 (as a checkpoint), 500, 600, 800, 1000, and 1200 μmol CO₂ mˉ²sˉ¹. In each *A*/*C*_i_ curve, the Amax represents the maximum rates of CO₂ uptake measured at high CO₂ concentrations and its corresponding *C*_i_ values.

The global atmospheric CO₂ concentration at the time this study was conducted was ∼400 ppm (<https://climate.nasa.gov/vital-signs/carbon-dioxide/>). Therefore, in each *A*/*C*_i_ curve, the net *CO*₂ assimilation (*A*) was measured at ambient *CO*₂ concentrations and its corresponding *C*_i_ values. Therefore, in the case of wild plants, the net *CO*₂ assimilation rate was measured to the ambient *CO*₂ concentration (i.e. R*CO*₂= 400 mol molˉ¹ and the corresponding *C*_i_ values), PPFD= 400 μmol mˉ²sˉ¹, and Tleaves= 32.7±0.9⁰C. In the case of plants grown in low sunlight, the net *CO*₂ assimilation rate was measured to the ambient CO₂ concentration (i.e. R*CO*₂= 400 mol molˉ¹ and the corresponding *C*_i_ values), PPFD= 94±0.4 μmol mˉ²sˉ¹, and Tleaves= 40± 2⁰C. Likewise, in the case of plants grown under intermediate sunlight, the net *CO*₂ assimilation rate was measured to the ambient *CO*₂ concentration (i.e. R*CO*₂= 400 mol molˉ¹ and the corresponding *C*_i_ values), PPFD= 648±4 μmol mˉ²sˉ¹, 3. Tleaves= 40± 2⁰C.

**GE curves fitting, parameters derivation, and additional ecophysiological traits assessment**

The measured *A*-PPFD curves and its parameters (i.e. *A*_max_, *R*_dark_, ɸ, LCP, PPFD₅₀, PPFD₉₅, *J*_max_) were solved (validated) by using different methods: Lobo *et al*. (2013), Sharkey (2016), and [www.landflux.org](http://www.landflux.org). The measured *A*/*C*_i_ curves were corrected to *A*/*C*_c_ curves by using the respective equation, removing by this way all diffusion resistance effects (Sun *et al*. 2014; Sharkey 2016, 2019). Afterward, we validated and parameterized the *A*/*C*_c_ curves to obtain the main photosynthetic parameters (i.e. *A*_max_, *A*, *V*_cmax_, *J*, *R*_d_, *g*_s_, T_r_, *g*_m_, *C*_ctr_). The *A*/*C*_c_ curves were solved by using two different curve fitting methods e.g. the linear and rectangular mathematical methods (Sun *et al*. 2014; Sharkey 2016). Both methods are based on the FvCB biophysical and biochemical model of photosynthesis and its general equations (Farquhar *et al*. 1980). To identify the photosynthetic plasticity traits, we compared the gas exchange parameters recorded in wild plants *vs* those recorded in cultivated plants under the different sunlight regimes. The trioses phosphate utilization rate (TPU) was solved using the tool proposed by Sharkey (2016). From the *A*/*C*_c_ curves, the maximum photosynthetic rates (*A*_max_) were measured to high CO₂ concentrations.

From the photosynthetic curves, the net *CO_2_* assimilation rates (*A*), stomatal conductance (*g*_s_), transpiration (*T_r_*), intercellular *CO*_2_ concentration (*C*_i_), and the chloroplastic *CO*_2_ concentration (*C*_c_), were measured under the following conditions: ambient *CO*₂ concentration (i.e. reference *CO*₂= 400 μmol molˉ¹), the irradiance at ambient reference levels (i.e. PPFD= 400, 94, 655 μmol mˉ²sˉ¹), and natural leaves temperature (*T*_leaves_= 33, 40, 42°C). These conditions were programmed for wild plants and for plants grown in the low and intermediate sunlight, respectively. The *g*_m_ was calculated using the respective equation (Sun *et al.* 2014; Sharkey 2016) and it was evaluated at the ambient *CO*₂ concentration (i.e. reference *CO*₂= 400 μmol molˉ¹). The ratio between *CO_2_* respiration and *CO_2_* assimilation (*R*_d_/*A* ratio) was derived from the curves at the ambient *CO₂* concentration. The rationale to take the ambient CO₂ concentration as reference (i.e. R*CO*₂= 400 μmol molˉ¹) for measure some key traits (e.g. *A*, *g*_s_, *t*_r_, *g*_m_, *C*_i_, *C*_c_, and *R*_d_/*A*), was based on the atmospheric *CO*_2_ concentration at the time of this study was conducted: ∼400 ppm.

The *CO*_2_ compensation and photocompensation points (*Г, Г*,* respectively) were solved with the approach proposed by Ethier and Livingston (2004) by using the fitting tool available in [www.landflux.org](http://www.landflux.org). The overall photosynthetic limitations were partitioned into three main functional components (i.e. stomatal: *l*_s_, mesophyll: *l*_m_, and biochemical: *l*_b_) by using the respective equations and the values of *g*_s_, *g*_m_, *V*_cmax_, *C*_i_, *C*_c_, *Г**, and Km derived from the *A*/*C*_i_ curves fitting (Martins *et al.* 2014; Sharkey 2016). The transition points (i.e. the *C*_ctr_ values) between the Rubisco and Rubp portions in photosynthetic curves were solved by the method M2c proposed by Sun *et al*. (2014). Immediately after completing the gas exchange measurements in cultivated plants, leaves were harvested and digitalized with a scanner to include them in total area quantification. Afterward, leaves were freezing (-20°C) until analysis of total photosynthetic pigments, chlorophylls a-b, xanthophylls, and carotenoids (Lichtenthaler and Buschmann 2001). The leaves' anatomical traits related to the diffusive capacity were evaluated under the optical microscope (Leica BX51) by counting the abaxial and adaxial stomatal density, as well as the abaxial stomatal index (Martins *et al*. 2014). The stomatal density and index are defined as the number of stomata per area unit and the ratio between the number of stomata and the number of surrounding epidermal cells, respectively. The stomatal density and the stomatal index were quantified in the center of the leaf lamina to a distance between 0.5-1 cm from the central vein.

For the determination of total photosynthetic pigments per unit of leaf area, we used the same leaves where gas exchange measurements were conducted. The chlorophylls were extracted in the dark using pure acetone (99.8%, Sigma Aldrich™) by macerating fresh fragments of leaves from plants grown under intermediate and low sunlight (2.5 mg fresh weight = 0.29±0.004 cmˉ², 2.5mg fresh weight = 0.15±0.004 cmˉ², respectively). The absorbance of extracts previously filtered was measured at different wavelengths (470, 644.8, and 661.6 nm). Then, the total photosynthetic pigments (TPP= *Chl*_a_, *Chl*_b_, *Xanth, Carot*) were determined according to the equations developed by Lichtenthaler and Buschmann (2001). To calculate the amount of nitrogen allocated to the main components of photosynthetic protein complexes (i.e. carboxylation, bioenergetics, light-harvesting), we applied the approach, equations, and constants proposed by Niinemets and Tenhunen (1997) and Yao *et al.* (2015). For such calculations, we used the data of leaf mass per area, the nitrogen content, the values of *V*_cmax_, *C*_c_, *J*, *C*_c_, and the total chlorophylls content (Niinemets *et al.* 2006; Yao *et al*. 2015). In such model equations, the Jmc is assumed as constant and represent the capacity for photosynthetic electron transport per unit cytochrome f (Jmc= 156 mol eˉ mol cyt f ˉ¹sˉ¹ at 25°C) (Niinemets and Tenhunen 1997); the Vcr is also assumed as a constant and represent the maximum rate of ribulose-1,5-bisphosphate carboxylation per unit Rubisco protein (Vcr = 20.5 μmol CO2 g Rubisco ˉ¹sˉ¹ at 25°C) (Niinemets and Tenhunen 1997).

The scaling coefficients 8.06 and 6.25 were used because they are constant parameters that are based on the stoichiometry of rate-limiting proteins and the nitrogen content of proteins (Niinemets and Tenhunen 1997). Although the actual nitrogen fractions invested in the photosynthetic apparatus may vary due to variability of the Rubisco specific activity and the rate of electron transport per unit cytochrome f, it is assumed that the values of Jmc and Vcr are constant, because they are used widely for study C₃ photosynthetic metabolism (Niinemets and Tenhunen 1997; Niinemets *et al.* 2006; Yao *et al*. 2015). In our analysis, the calculated values of nitrogen allocation to photosynthetic components were proportional and consistent with the total fraction of N per unit of leaf area; therefore, they constitute a general representation of the rate-limiting proteins complexes of photosynthesis (Niinemets and Tenhunen 1997; Niinemets *et al*. 2006).

**Plants harvests, growth rate, and biomass allometry assessment**

After the gas exchange measurements were conducted, all leaves from each plant (n= 6 plants per treatment; between 170-750 leaves per plant were harvested) were harvested and digitalized. Afterward, with the ImageJ software (<https://imagej.nih.gov/ij/download.html>), the individual leaf area (ILA= cm²) and the leaf area index (LAI= m²mˉ²) were quantified. Afterward, to remove moisture remnants, each organ (i.e. roots, stems, leaves, fruits) was dried to 45°C for 24 h and then to 40°C for another 24 h (just in the case of roots, stems, and fruits). By harvesting six plants (n= 6) from the low sunlight regimes and six plants from the intermediate sunlight regimes, we assessed plants' growth, their architecture, and biomass allometry through an analysis of different parameters: 1. The relative growth rates (RGR), 2. Leaf mass per area (LAI), 3. Individual leaf area (ILA), 4. Specific leaf area (SLA), 5. Leaf mass per area (LMA), 6. Leaf area ratio (LAR), 7. Total dry biomass (TB), 8. Shoot/Roots ratio (S/R ratio), 9. Roots mass fraction (RMF), 10. Stems mass fraction (SMF), 11. Leaf mass fraction (LMF) and 12. Specific stem length (SSL) (Poorter *et al*. 2012, 2015). The reproductive yield was quantified by measuring the fruits' fresh weight (FFW), fruit's dry weight (FDW), and reproductive mass fraction (REMF) on a dry basis. The dried leaves from each harvested plant were used for the determination of macroelements and microelements (i.e. C, H, N, P, S, Fe, Ni, Cu, Na, K, Mn, Mg, Zn).

**Elemental analysis**

All canopy leaves from harvested plants were dried, grounded to a fine powder, and then subsamples were used to quantify the content of carbon (C), hydrogen (H), nitrogen (N), and sulfur (S), by using the organic elemental analysis protocol (FLASH 2000 analyzer, Thermo Scientific™). Other subsamples were digested with concentrated HNO₃ (65%) and H₂O₂ (30%). Afterward, the content of phosphorus (P) was determined by the molybdenum-vanadate method using a UV–vis spectrophotometer; and the contents of potassium (K), sodium (Na), calcium (Ca), magnesium (Mg), manganese (Mn), zinc (Zn), iron (Fe), nickel (Ni), and copper (Cu), were determined by atomic absorption spectroscopy (Thermo Scientific™). By using the respective calibration curves (R² >0.99) and its derived equations, elements concentration was determined. Afterward, the total content of macroelements and microelements was calculated on a leaf area basis.

**Results**

**
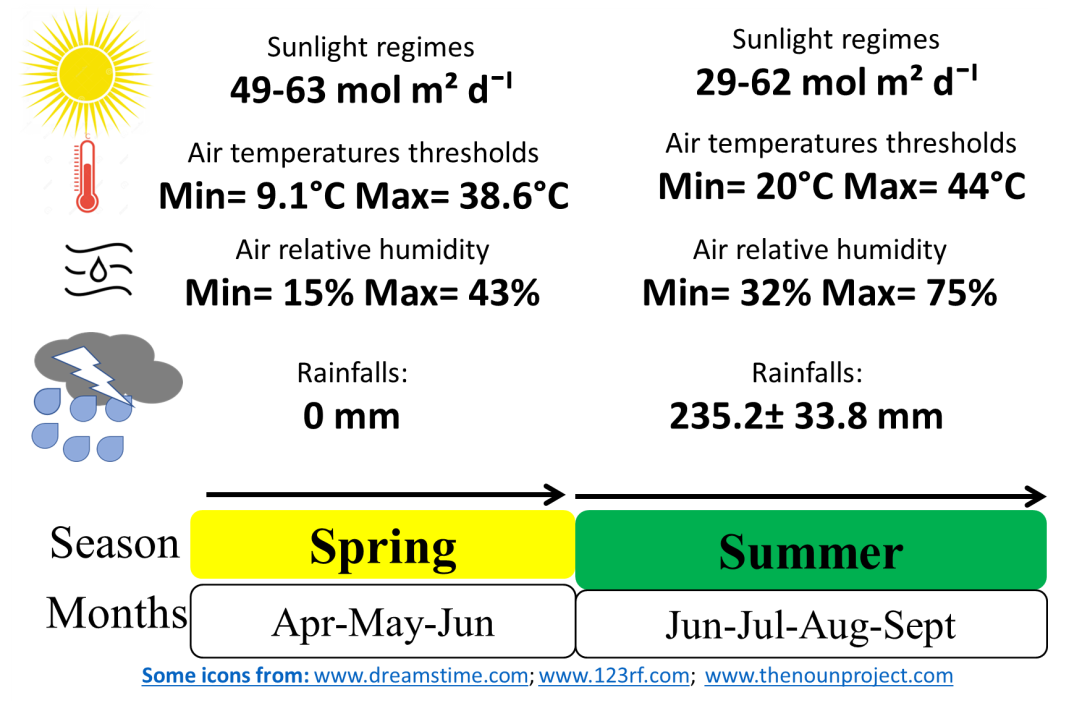
**

**Figure S2.** Summary of weather conditions recorded at the open sky (i.e. outside of the shading nets) from seeds sowing in mid-spring, and throughout the summer. Data reflect the seasonal minimum and maximum thresholds recorded. Dataset was recorded during 157 days of experimentation in 2016. Sunlight regimes were recorded with a WatchDog MicroStation. The records of air temperatures, air relative humidity, and accumulated rainfalls were obtained from (<http://www.siafeson.com/remas2/index.php/tablero>).

**Table S2.** Sunlight regimes (SLR= PPFD) and air temperatures thresholds recorded during cultivation of plants. Spring: the values at open sky *vs* the values at the intermediate and low sunlight are shown. Summer: the values at open sky *vs* the values at the high, intermediate, and low sunlight are shown.

| Phenological stage | Season | Sunlight regimes | Photosynthetic photons flux density PPFD | | | |  | Air temperature thresholds (°C) | | |
| --- | --- | --- | --- | --- | --- | --- | --- | --- | --- | --- |
|  |  |  | Imax µmol m² sˉ¹ | Imin µmol m² sˉˡ | Dmax mol m² dˉˡ | Dmin mol m² dˉˡ |  | Maximum | Minimum | Averages |
|  |  |  |  |  |  |  |  |  |  |  |
| From seeds sowing until early vegetative development | Spring | Open sky | 2289±96 a | 9.6±1 a | 63±3 a | 49±10 a | Spring | 38.6±2.3 a | 9.1±1.02 a | 23.8±1.7 a |
|  |  | Intermediate | 621±26 b | 10.±1.5 a | 15.3±0.5 b | 10.6± 2 b |  | 46.1±7.6 a | 11.6±0.06 a | 28.9±3.8 a |
|  |  | Low | 92± 9 c | 9± 0.5 a | 2 c | 1.6± 0.5 c |  | 49.8±0.3 a | 9.9±1.2 a | 29.9±0.4 a |
| After transplant until full vegetative and reproductive development | Summer | Open sky | 2328±141 a | 12±3 a | **62±4 a** | 29±12 a | Summer | **44.2±2.5 a** | 20.1±1.6 a | 32.2±0.7 a |
|  |  | High | 1906±88 b | 11±3 a | **46±5 b** | 21±8 b |  | **61±2.1 b** | 18.4±3.5 a | 39.7±1 b |
|  |  | Intermediate | 680±57 c | 9±0.9 a | **15±1.5 c** | 7±2.7 c |  | **56.4±0.9 c** | 19±3 a | 37.8±1.3 b |
|  |  | Low | 108±18 d | 9±0.5 a | **2±0.5 d** | 0.7±0.5 d |  | **49.3±2 d** | 19±3 a | 32.6±1.7 a |

Averages contrast is by column according to season. Averages with different letters were significant p˂0.05 by ANOVA-Fisher LSD test. Imin, instantaneous minimum, Imax, instantaneous maximum, Dmin, daily minimum, Dmax, daily maximum. Spring: April, May, June. Summer: June, July, August, September.

**Table S3.** Summary of soil temperatures recorded at each sunlight exposure level during plants cultivation.

| Season | Sunlight regimes | Soil temperatures °C |
| --- | --- | --- |
| Summer | high | 38 ± 1.4 |
|  | Intermediate | 33 ± 0.2 |
|  | Low | 30 ± 0.2 |

**Table S4.** Results of analysis of variance of photosynthetic parameters obtained from A-PPFD curves measured in wild and cultivated plants in low and intermediate sunlight (p˂0.05).

| Measurements | Photosynthetic parameters | | | | | | | | | | |
| --- | --- | --- | --- | --- | --- | --- | --- | --- | --- | --- | --- |
|  | Tleaf °C | VPD kPa | RH % | Rd µmol CO₂  mˉ² sˉ¹ | Initial slope ɸ | LCP µmol CO₂  mˉ² sˉ¹ | PPFD₅₀ µmol mˉ² sˉ¹ | PPFD₉₅ µmol mˉ² sˉ¹ | Amax µmol CO₂  mˉ² sˉ¹ | Curvature Ꝋ | Jmax µmol e mˉ² sˉ¹ |
| Wild  Cultivated plants in low sunlight  Cultivated plants under intermediate sunlight | **0** | **0** | **0** | **0.0213** | 0.2995 | **0.0002** | 0.369 | **0.0344** | **0.0004** | 0.1557 | **0.006014** |

**Table S5.** Photosynthetic parameters derived from the *A*/*C*_c_ curves fitting, using the M1c (linear) and ST (rectangular) methods (Sun *et al*. 2014; Sharkey 2016). The parameters correspond to wild plants (as reference values) and cultivated plants in low and intermediate sunlight regimes.

| Parameters | Wild plants | Cultivated Plants | |
| --- | --- | --- | --- |
|  |  | Treatments | |
|  |  |  |  |
|  |  | Low sunlight | Intermediate sunlight |
| M1c, *V*_cmax_ *Tleaf* (µmol CO₂ mˉ²sˉ¹) | 117±36^a^ | 50±13^b^ | 99±27^a^ |
| M1c, *V*_cmax_ *adj25* (µmol CO₂ mˉ²sˉ¹) | 54±11^a^ | 15±1^b^ | 24±5^c^ |
| M1c, *J* *Tleaf* (µmol eˉ mˉ²sˉ¹) | 94±14^a^ | 40±2^b^ | 64±6^c^ |
| M1c, *J* *adj25* (µmol eˉ mˉ²sˉ¹) | 60±9^a^ | 18±2^b^ | 25±3^c^ |
| *J* /Vcmax | 0.83±0.1^a^ | 0.85±0.1^a^ | 0.67±0.09^b^ |
| M1c, *R*_d_ *Tleaf* (µmol CO₂ mˉ² sˉ¹) | 1.2±0.2^a^ | 2±0.3^b^ | 4±0.9^c^ |
| M1c, *R*_d_ *adj25* (µmol CO₂ mˉ² sˉ¹) | 0.7^a^ | 0.9±0.09^a^ | 1.4±0.3^b^ |
| ST, *V*_cmax_ *Tleaf* (µmol CO₂ mˉ²sˉ¹) | 102±39^a^ | 53±11^b^ | 96±37^a^ |
| ST, *V*_cmax_ *adj25*(µmol CO₂ mˉ²sˉ¹) | 51±16^a^ | 15±0.9^b^ | 24±8^c^ |
| ST, *J Tleaf* (µmol eˉ mˉ²sˉ¹) | 93±17^a^ | 41±2^b^ | 65±12^c^ |
| ST, *J adj25* (µmol eˉ mˉ²sˉ¹) | 59±11^a^ | 18±4^b^ | 26±4^c^ |
| *J* /*Vcmax* | 0.95±0.1^a^ | 0.80±0.1^a^ | 0.73±0.1^a^ |
| ST, *R*_d_ *Tleaf* (µmol CO₂ mˉ² sˉ¹) | 1.5±0.48^a^ | 2.5±0.2^b^ | 4.5±1^c^ |
| ST, *R*_d_ *adj25* (µmol CO₂ mˉ² sˉ) | 0.9±0.2^a^ | 1.0±0.1^a^ | 1.7±0.5^b^ |

Averages contrast is by row. Averages with different letters were significant p˂0.05 by a one way ANOVA-Fisher LSD test. Tleaf: parameters derived at leaf temperatures. Parameters at 25°C (adj25) were calculated by using the respective equations and the temperature dependent kinetic parameters for Rubisco (25°C) described on a Cc basis (Sun *et al*. 2014; Sharkey 2016).

**Table S6.** Results of the analysis of variance of the photosynthetic traits measured on wild and cultivated plants in low and intermediate sunlight regimes. *p˂0.05 (n= 6).

| Parameters | Wild plants | Cultivated Plants | |
| --- | --- | --- | --- |
|  |  | Treatments | |
|  |  |  |  |
|  |  | Low sunlight | Intermediate sunlight |
| Air temperatures | 0.000758* | | |
| PPFD (µmol e mˉ²sˉ¹) | 0.0000* | | |
| Tleaf °C | 0.000718* | | |
| VPD kPa | 0.05336 | | |
| *g_s_* (mmol H₂O mˉ²sˉ¹) | 0.007855* | | |
| *t_r_* (mmol H₂O mˉ²sˉ¹) | 0.008850* | | |
| *A/g_s_* (µmol CO₂ mol H₂O) | 0.000000* | | |
| *A/t_r_* (µmol CO₂ mmol H₂O) | 0.000004* | | |
| *g_m_* (µmol CO₂ mˉ²sˉ¹) | 0.019093* | | |
| *Cᵢ* (µmol CO₂ molˉ¹ air) | 0.000001* | | |
| *C_c_* (µmol CO₂ molˉ¹ air) | 0.000004* | | |
| *g_m_*/*g_s_ ratio*(mol CO₂ molˉ¹ CO₂) | 0.006081* | | |
| Stomatal limitation *l*_s_ | 0.000012* | | |
| Mesophyll limitation *l*_m_ | 0.000127* | | |
| Biochemical limitation *l*_b_ | 0.004505* | | |
| *A_max_* (µmol CO₂ mˉ²sˉ¹) | 0.000007* | | |
| *A* (µmol CO₂ mˉ²sˉ¹) | 0.000260* | | |
| *R*_d_/*A* ratio (µmol CO₂/µmol CO₂ mˉ²sˉ¹) | 0.005434* | | |
| *V_cmax_* (µmol CO₂ mˉ²sˉ¹) | 0.037527* | | |
| *J* (µmol eˉ mˉ²sˉ¹) | 0.000178* | | |
| *J*/ *V_cmax_* ratio | 0.561338 | | |
| *C_ctr_* (Pa) | 0.002655* | | |
| TPU (µmol CO₂ mˉ²sˉ¹) | 0.000024* | | |
| *R_d_* (µmol CO₂ mˉ²sˉ¹) | 0.000569* | | |
| *Г* (µmol molˉ¹) | 0.000001* | | |
| *Г** (µmol molˉ¹) | 0.003659* | | |


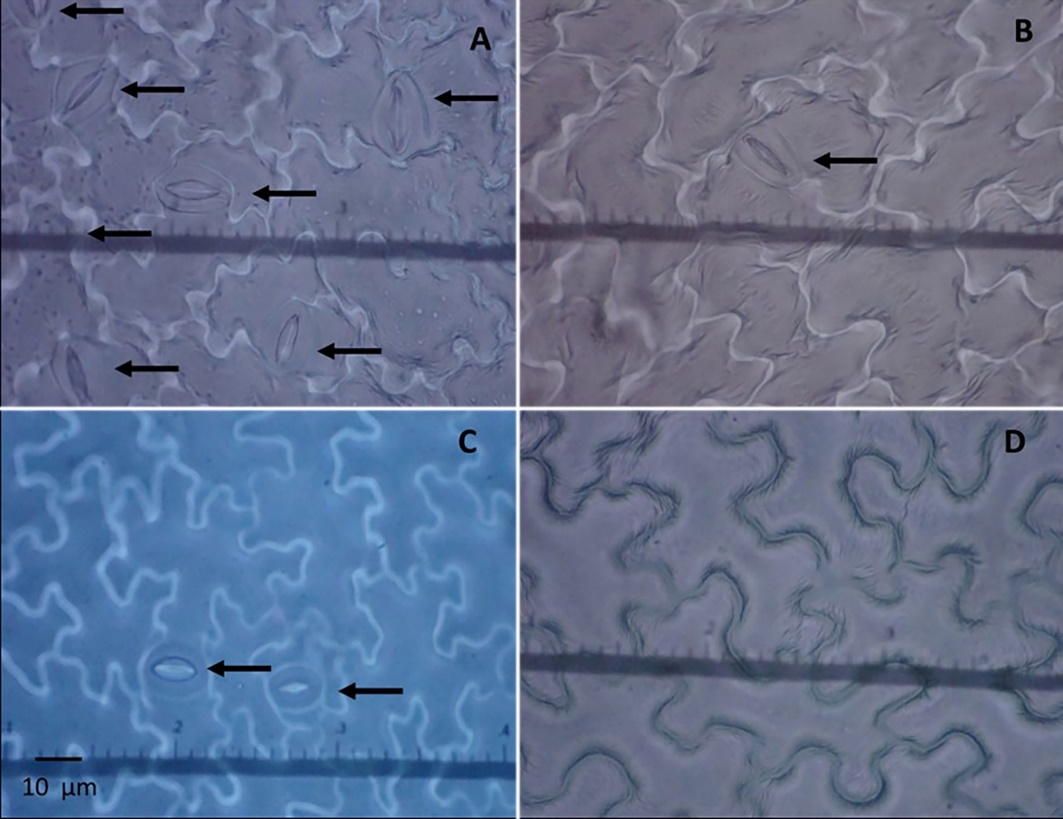


**Figure S3.** Stomatal density on the abaxial and adaxial surface of leaves from plants grown under intermediate (A, B) and low (C, D) sunlight regimes.

**Table S7.** Results of the analysis of variance of architectural, anatomical, and biochemical traits from plants grown in low and intermediate sunlight regimes * p˂0.05 (n= 6).

| Parameters | Cultivated Plants | |
| --- | --- | --- |
|  | Treatments | |
|  |  |  |
|  | Low sunlight regimes | Intermediate sunlight regimes |
| Specific leaf area (SLA= m² gˉ¹) | 0.0000* | |
| Leaf area ratio (LAR= m² gˉ¹) | 0.0000* | |
| Individual leaf area (ILA= cm²) | 0.0000* | |
| Leaf mass per area (LMA= g mˉ²) | 0.0000* | |
| Adaxial stomatal density per cm² | 0.0000* | |
| Abaxial stomatal density per *cm² | 0.0000* | |
| Stomatal index (%) | 0.0005* | |
| Total photosynthetic pigments (TPP= g mˉ²) | 0.000000* | |
| Chlorophyll a (Chla= mg mˉ²) | 0.000001* | |
| Chlorophyll b (Chlb= mg mˉ²) | 0.000008* | |
| Xanthophylls and carotenoids (X+C= mg mˉ²) | 0.000010* | |
| Nitrogen (N= g mˉ²) | 0.079091 | |
| Nitrogen to carboxilation (NC= mg g N mˉ²) | 0.01869* | |
| Nitrogen to bioenergetics (NB= mg g N mˉ²) | 0.004893* | |
| Nitrogen to light harvesting (NL= mg g N mˉ²) | 0.00005* | |
| Nitrogen to photosynthetic components (NP= mg g N mˉ²) | 0.00025* | |
| NL/NP (mg gˉ¹) | 0.006485* | |
| Carbon (g mˉ²) | 0.000078* | |
| Sulphur (S= g mˉ²) | 0.002913* | |
| Hydrogen (H= g mˉ²) | 0.000019* | |
| Phosphorus (P= g mˉ²) | 0.002698* | |
| Magnesium (Mg= g mˉ²) | 0.000705* | |
| Calcium (Ca= g mˉ²) | 0.692982 | |
| Potasium (K= g mˉ²) | 0.031786* | |
| Sodium (Na= g mˉ²) | 0.469107 | |
| Manganesum (Mn= mg mˉ²) | 0.819614 | |
| Iron (Fe= mg mˉ²) | 0.012906* | |
| Nickel (Ni= mg mˉ²) | 0.000026* | |
| Zinc (Zn= mg mˉ²) | 0.361888 | |
| Cooper (Cu= mg mˉ²) | 0.002951* | |

**Table S8.** Results of the analysis of variance of growth parameters from plants grown in low and intermediate sunlight regimes * p˂0.05 n= 6.

| Traits | Treatments | |
| --- | --- | --- |
|  |  |  |
|  | Low sunlight regimes | Intermediate sunlight regimes |
| Relative growth rate (RGR= g gˉ¹ dˉ¹) | *0.001689 | |
| Total biomass (TB= g) | *0.028109 | |
| Leaves area index (LAI= m² mˉ²) | 0.0000* | |
| Fruits fresh weight (FFW= g) | *0.000495 | |
| Fruits dry weight (FDW= g) | *0.0038 | |
| Shoots/Roots ratio (S/R ratio= g gˉ¹) | *0.001467 | |
| Roots mass fraction (RMF= g gˉ¹) | *0.000902 | |
| Stems mass fraction (SMF= g gˉ¹) | 0.151566 | |
| Leaves mass fraction (LMF= g gˉ¹) | *0.000068 | |
| Reproductive mass fraction (REMF= g gˉ¹) | *0.001995 | |
| Specific stem lenght (SSL= cm gˉ¹) | *0.001518 | |

Multiple correlations between measured traits were recorded (see Supporting Information B). The stomatal conductance (*g_s_*) positively correlated to intercellular *CO_2_* concentration (*C_i_*), and negatively correlated to stomatal limitation (*l_s_*) (see Supporting Information B). The stomatal limitation (*l*_s_) positively correlated to *A*/*g*_s_ and negatively to the stomatal conductance (*g_s_*). The mesophyll conductance (*g_m_*) positively correlated to the ratio between the mesophyll and stomatal conductance (*g_m_/g_s_* ratio), moderately correlated to chloroplastic *CO_2_* concentration (*C_c_*), and negatively correlated to mesophyll limitation (*l_m_*). The chloroplastic *CO_2_* concentration (*C_c_*) negatively correlated to photosynthetic capacity traits (*V_cmax_*, *J*, and TPU), respiration (*R_d_*), and mesophyll limitation (*l_m_*). The mesophyll limitation (*l*_m_) positively correlated to some parameters of photosynthetic capacity (*J*, TPU), respiration (*R*_d_), as well as to nitrogen allocation to carboxylation and bioenergetics (NC, NB). The ratio between respiration and assimilation (*R_d_*/*A*) positively correlated to the Rubisco carboxylation velocity (*V_cmax_*), and the reproductive mass fraction (REMF). The maximum *CO_2_* assimilation (*A_max_*), Rubisco carboxylation velocity (*V_cmax_*), electrons transport (*J*), trioses phosphate utilization (TPU), and respiration (*R_d_*), positively correlated to each other; but also they correlated to several biochemical traits (e.g. TPP, *Chl*_a_, *Chl*_b_, C, H, NL, NP), abaxial stomatal density, and growth traits (LMA, RGR, TB) (see Supporting Information B).

The specific leaf area (SLA) and leaf area ratio (LAR) were negatively correlated to photosynthetic capacity traits (A_max_, V_cmax_, J, TPU), respiration (Rd), the content of photosynthetic pigments (TPP, *Chl_a_, Chl_b_*, *Xant*, *Carot*), nitrogen allocation to photosynthetic components (NC, NB, NL), leaf mass per area (LMA), abaxial and adaxial stomatal density (*SD_ab_*, *SD_ad_*), stomatal index (*SI_ab_*), carbon, hydrogen, mineral nutrients, and growth traits (see Supporting Information B). The leaf mass per area positively correlated to maximum assimilation (*A_max_*), the abaxial and adaxial stomatal density (SD_ab_), the stomatal index (SI_ab_), content of photosynthetic pigments (TPP, *Chl_a_, Chl_b_*, *Xant*, *Carot*), nitrogen allocation to light harvesting (NL, NL/NP), carbon, hydrogen, magnesium, nickel. The adaxial and abaxial stomatal density (*SD_ad_*, *SD_ab_*), as well as the stomatal index (*SI_ab_*), were positively correlated to the leaf mass per area (LMA), content of photosynthetic pigments (TPP, *Chl_a_, Chl_b_*, *Xant*, *Carot*), and nitrogen allocation to light harvesting protein complex (*NL*). The content of photosynthetic pigments (TPP, *Chl_a_, Chl_b_*, *Xant*, *Carot*) positively correlated to several traits e.g. the maximum assimilation (Amax), trioses phosphate utilization (TPU), the leaf mass per area (LMA), the abaxial and adaxial stomatal density (*SD_ab_*), the stomatal index (SI_ab_), nitrogen allocation to light harvesting complexes (NL), carbon and hydrogen content (C, H), mineral nutrients content (P, Mg, Ni, Cu). The nitrogen allocation to carboxylation and bioenergetics (NC, NB) positively correlated to several traits e.g. photosynthetic capacity (V_cmax_, *J*, TPU), respiration (*R_d_*), and mesophyll limitation (l_s_). The nitrogen allocation to carboxylation positively correlated to the relative growth rate, total biomass, and reproductive mass fraction (see Supporting Information B).

The nitrogen allocation to the light harvesting (NL, NL/NP) positively correlated to the maximum assimilation (*Amax*), the trioses phosphate utilization (TPU), the pigments content (TPP, *Chl_a_, Chl_b_*, *Xant*, *Carot*), the leaf mass per area (LMA), the abaxial and adaxial stomatal density (*SD_ab_*), the stomatal index (SI_ab_), carbon, hydrogen, and some minerals (P, Mg, Ni, Cu) (see Supporting Information B). The total nitrogen allocated to photosynthetic components (NP) positively correlated to several traits e.g. photosynthetic capacity (A_max_, Vcmax, J, TPU), respiration (Rd), mesophyll limitation (ls), the leaf mass per area (LMA), the pigments content (TPP, *Chl_a_, Chl_b_*), carbon, hydrogen, and some minerals (S, P, Mg, Ni, Cu), and growth (RGR, TB, REMF). The carbon and hydrogen content (C, H) positively correlated to several traits e.g. photosynthetic capacity traits (A_max_, *J*, TPU), respiration (*R_d_*), pigments content (TPP, *Chl_a_, Chl_b_*, *Xant*, *Carot*), mineral nutrients content (S, P, Mg, Ni, Cu), nitrogen allocation to photosynthetic components (NB, NL, NL/NP, NP), abaxial and adaxial stomatal density (*SD_ab_*), and leaf mass per area (LMA). The iron content (Fe) positively correlated to phosphorus and copper content (P, Cu). The magnesium content (Mg) positively correlated to photosynthetic pigments (TPP, Chla, Chlb) and total nitrogen allocation to photosynthetic components (NP). Several nutrients (S, P, Mg, Ca, Ni, Cu) positively correlated to total nitrogen allocation to photosynthetic components. The relative growth rate and total biomass positively correlated to specific photosynthetic capacity traits (*V*_cmax_, *J*) and nitrogen allocation to carboxylation and nitrogen allocation to total photosynthetic components (NC, NP). The reproductive mass fraction positively correlated to different photosynthetic capacity traits (*R_d_/A* ratio, *V*_cmax_, *J*, TPU, *R_d_*), nitrogen allocation to carboxylation and nitrogen allocation to total photosynthetic components (NC, NP) (see Supporting Information B).

**Literature cited in this Supporting Information**

CONAGUA 2014. National Water Commission of Sonora State in México. Historical climatological data from the Hermosillo Sonora Climatological Observatory Station. Personal Communication and consultation of the database.

Ethier GJ, Livingston NJ. 2004. On the need to incorporate sensitivity to CO_2_ transfer conductance into the Farquhar–von Caemmerer–Berry leaf photosynthesis model. *Plant Cell and Environment* 27:137–153.

Farquhar GD, von Caemmerer S, Berry JA. 1980. A biochemical-model of photosynthetic CO_2_ assimilation in leaves of C_3_ species. *Planta* 149:78–90.

Jiménez-Leyva JA, Gutiérrez A, Orozco JA, Vargas G, Esqueda M, Gardea A, González-Hernández V, Sánchez E, Muñoz E. 2017. Phenological and ecophysiological responses of *Capsicum annuum* var. *glabriusculum* to native arbuscular mycorrhizal fungi and phosphorus availability. *Environmental and Experimental Botany* 138:193–202.

Lichtenthaler HK, Buschmann C. 2001. Chlorophylls and carotenoids: Measurement and characterization by UV-VIS spectroscopy. In: Wrolstad RE, Acree TE, An H, Decker EA, Penner MH, Reid DS, et al., eds, Current Protocols in Food Analytical Chemistry. New York: John Wiley and Sons, F4.3.1-F4.3.8.

Lobo FA, de Barros MP, Dalmagro HJ, Dalmolin ÂC, Pereira WE, de Souza EC, Vourlitis GL, Rodríguez CE. 2013. Fitting net photosynthetic light-response curves with Microsoft Excel - a critical look at the models. *Photosynthetica* 51:445–456.

Martins SCV, Galmes J, Cavatte PC, Pereira LF, Ventrella MC, DaMatta FM. 2014. Understanding the low photosynthetic rates of sun and shade coffee leaves: Bridging the gap on the relative roles of hydraulic, diffusive, and biochemical constraints to photosynthesis. *PLoS ONE* 4:e95571.

Moreno LSB, Pedreira CGS, Boote KJ, Alves RR. 2014. Base temperature determination of tropical *Panicum* spp. grasses and its effects on degree-day-based models. *Agricultural and Forest Meteorology* 186:26–33.

Niinemets U, Tenhunen J. 1997. A model separating leaf structural and physiological effects on carbon gain along light gradients for the shade-tolerant species *Acer saccharum*. *Plant Cell and Environment* 20:845–866.

Niinemets U, Cescatti A, Rodeghiero M, Tosens T. 2006. Complex adjustments of photosynthetic potentials and internal diffusion conductance to current and previous light availabilities and leaf age in Mediterranean evergreen species *Quercus ilex*. *Plant Cell and Environment* 29(6):1159–1178.

Poorter H, Niklas KJ, Reich PB, Oleksyn J, Poot P, Mommer L. 2012. Biomass allocation to leaves, stems and roots: meta-analyses of interspeciﬁc variation and environmental control. *New Phytologist* 193:30–50.

Poorter H, Jagodzinski AM, Peinado RR., Kuyah S, Luo Y, Oleksyn J, Usoltsev VA, Buckley TN, Reich PB, Sack L. 2015. How does biomass distribution change with size and differ among species? An analysis for 1200 plant species from ﬁve continents. *New Phytologist* 208:736–749.

Rodeghiero M, Niinemets U, Cescatti A. 2007. Major diffusion leaks of clamp-on leaf cuvettes still unaccounted: how erroneous are the estimates of Farquhar et al. model parameters? *Plant Cell and Environment* 30(8):1006–1022.

Sharkey TD. 2016. What gas exchange data can tell us about photosynthesis. *Plant Cell and Environment* 39:1161–1163.

Sharkey TD. 2019. Is triose phosphate utilization important for understanding photosynthesis? *Journal of Experimental Botany* 70:5521–5525.

Sun J, Feng Z, Leakey ADB, Zhu X, Bernacchi CJ, Ort DR. 2014. Inconsistency of mesophyll conductance estimate causes the inconsistency for the estimates of maximum rate of Rubisco carboxylation among the linear, rectangular, and non-rectangular hyperbola biochemical models of leaf photosynthesis - A case study of CO_2_ enrichment and leaf aging effects in soybean. *Plant Science* 226:49–60.

Yao H, Zhang Y, Yi X, Hu Y, Luo H, Gou L, Zhang W. 2015. Plant density alters nitrogen partitioning among photosynthetic components, leaf photosynthetic capacity, and photosynthetic nitrogen use efficiency in field-grown cotton. *Field Crops Research* 184:39–49.
